# The three-way junction structure of the HIV-1 PBS-segment binds host enzyme important for viral infectivity

**DOI:** 10.1093/nar/gkab342

**Published:** 2021-05-12

**Authors:** Zhenwei Song, Thomas Gremminger, Gatikrushna Singh, Yi Cheng, Jun Li, Liming Qiu, Juan Ji, Margaret J Lange, Xiaobing Zuo, Shi-Jie Chen, Xiaoqin Zou, Kathleen Boris-Lawrie, Xiao Heng

**Affiliations:** Department of Biochemistry, University of Missouri, Columbia, MO, 65211, USA; Department of Biochemistry, University of Missouri, Columbia, MO, 65211, USA; Department of Veterinary and Biomedical Sciences, University of Minnesota, Saint Paul, MN 55108, USA; Department of Biochemistry, University of Missouri, Columbia, MO, 65211, USA; Department of Physics and Astronomy, University of Missouri, Columbia, MO 65211, USA; Institute for Data Science and Informatics, University of Missouri, Columbia, MO 65211, USA; Department of Biochemistry, University of Missouri, Columbia, MO, 65211, USA; Department of Physics and Astronomy, University of Missouri, Columbia, MO 65211, USA; Institute for Data Science and Informatics, University of Missouri, Columbia, MO 65211, USA; Department of Biochemistry, University of Missouri, Columbia, MO, 65211, USA; Department of Physics and Astronomy, University of Missouri, Columbia, MO 65211, USA; Institute for Data Science and Informatics, University of Missouri, Columbia, MO 65211, USA; Dalton Cardiovascular Research Center, University Missouri, Columbia, MO 65211, USA; Department of Biochemistry, University of Missouri, Columbia, MO, 65211, USA; Department of Molecular Microbiology and Immunology, University of Missouri, Columbia, MO 65211, USA; X-Ray Science Division, Argonne National Laboratory, Lemont, IL, 60439, USA; Department of Biochemistry, University of Missouri, Columbia, MO, 65211, USA; Department of Physics and Astronomy, University of Missouri, Columbia, MO 65211, USA; Institute for Data Science and Informatics, University of Missouri, Columbia, MO 65211, USA; Department of Biochemistry, University of Missouri, Columbia, MO, 65211, USA; Department of Physics and Astronomy, University of Missouri, Columbia, MO 65211, USA; Institute for Data Science and Informatics, University of Missouri, Columbia, MO 65211, USA; Dalton Cardiovascular Research Center, University Missouri, Columbia, MO 65211, USA; Department of Veterinary and Biomedical Sciences, University of Minnesota, Saint Paul, MN 55108, USA; Department of Biochemistry, University of Missouri, Columbia, MO, 65211, USA

## Abstract

HIV-1 reverse transcription initiates at the primer binding site (PBS) in the viral genomic RNA (gRNA). Although the structure of the PBS-segment undergoes substantial rearrangement upon tRNA^Lys3^ annealing, the proper folding of the PBS-segment during gRNA packaging is important as it ensures loading of beneficial host factors. DHX9/RNA helicase A (RHA) is recruited to gRNA to enhance the processivity of reverse transcriptase. Because the molecular details of the interactions have yet to be defined, we solved the solution structure of the PBS-segment preferentially bound by RHA. Evidence is provided that PBS-segment adopts a previously undefined adenosine-rich three-way junction structure encompassing the primer activation stem (PAS), tRNA-like element (TLE) and tRNA annealing arm. Disruption of the PBS-segment three-way junction structure diminished reverse transcription products and led to reduced viral infectivity. Because of the existence of the tRNA annealing arm, the TLE and PAS form a bent helical structure that undergoes shape-dependent recognition by RHA double-stranded RNA binding domain 1 (dsRBD1). Mutagenesis and phylogenetic analyses provide evidence for conservation of the PBS-segment three-way junction structure that is preferentially bound by RHA in support of efficient reverse transcription, the hallmark step of HIV-1 replication.

## INTRODUCTION

The HIV-1 5′ untranslated region (5′UTR) consists of a series of structural elements that coordinate various viral replication events. The hallmark event of reverse transcription initiates on the 5′UTR by annealing of tRNA^Lys3^ with the complementary primer binding site (PBS) (1). Annealing does not occur spontaneously at physiological temperature, as both the PBS-containing segment (PBS-segment) of viral RNA and tRNA^Lys3^ are highly structured. Complete annealing requires mature nucleocapsid (NC) protein, which is produced after maturation of the virion proteins (2–5).

Several discrete regions in the PBS-segment are known to interact with viral and host factors to promote reverse transcription (6–14). The primer activation signal (PAS) has been reported to promote tRNA priming and enhance reverse transcription efficiency (6–9), but it is under debate whether PAS directly interacts with tRNA^Lys3^ during reverse transcription initiation (15). Lysyl-tRNA synthetase (LysRS) has been reported to facilitate the annealing step by placing tRNA^Lys3^ onto the PBS-segment. The process is mediated by the interactions between the anticodon binding domain of LysRS and the tRNA-like element (TLE) within the PBS-segment (10), which mimics the tRNA^Lys3^ anticodon for LysRS binding and increases the efficiency of tRNA^Lys3^ annealing (11–14).

DHX9/RNA helicase A (RHA) co-assembles with the HIV-1 genomic RNA (gRNA) and bolsters virion infectivity (16,17). RHA belongs to the DExH-box superfamily and unwinds RNA duplex in the 3′- to 5′- direction. It has two double-stranded RNA binding domains (dsRBDs) at the N-terminus, followed by two RecA-like domains in the core helicase region, and an RG-rich domain on the C-terminus. Both dsRBDs are required for RHA to interact with the 5′UTR (16–18). A point mutation that disrupted the structure of the PBS-segment was deficient in interacting with the N-terminus domain of RHA (dsRBD1+dsRBD2) and resulted in reduced infectivity (19). RHA does not re-arrange the annealed RNA complex for reverse transcription initiation, but enhances processivity of reverse transcriptase (RT) during the elongation phase by unwinding the RNA secondary and/or tertiary structures (20).

Hence, prior to tRNA^Lys3^ annealing, the PBS-segment RNA serves as a scaffold to interact with viral and host factors, including aforementioned NC, tRNA^Lys3^, LysRS, RHA and possibly other yet to be characterized factors (6–14). These interactions may be mechanistically linked to achieve appropriate primer annealing for the temporal control of reverse transcription initiation. Chemical probing, enzymatic probing, computational modeling, as well as recent small angle X-ray scattering (SAXS) data supported formation of the TLE stem loop and PAS stem in the PBS-segment, connected by a long flexible loop that encompasses the tRNA^Lys3^ annealing site (21–25). However, these structural features leave gaps in knowledge of structural basis for the recruitment of some factors, including RHA. Lack of high-resolution structural information of the PBS-segment has hampered the structure-function investigation of the RNA: protein interactions necessary for efficient reverse transcription initiation.

Here, we report the solution structure of the HIV-1 PBS-segment RNA and its crucial role in RNA helicase interaction necessary for efficient reverse transcription. The results characterize a previously unidentified three-way junction structure that is composed of the TLE, tRNA annealing arm and PAS. Phylogenetic analysis of HIV-1 clinical sequences documents that the secondary structure was highly conserved among various HIV-1 strains, and computational mutagenesis scanning analysis shows that the base pairings in the bottom of the TLE stem are necessary to maintain the three-way junction structure. SAXS data of PBS-segment mutant RNAs demonstrate that disruption of base pairings at the bottom of TLE stem altered RNA folding, and reduced infectivity of progeny viruses attributable to deficient recruitment of RHA to the PBS-segment, as would be expected based on previous results (19). Combining electrophoretic mobility shift assay (EMSA), isothermal titration calorimetry (ITC), NMR and advanced molecular docking, we show that the three-way junction structure of the PBS-segment is specifically recognized by RHA. RHA’s dsRBD1 and dsRBD2-Core domains are likely to manifest the selective shape recognition mechanism. In summary, our data demonstrated that the three-way junction structure of the PBS-segment structure is conserved to support infectivity of progeny viruses.

## MATERIALS AND METHODS

### Plasmids

The PBS-segment, 5′UTR, 5′UTR^ΔTAR-PolyA^ and 5′UTR^ΔPBS^ plasmids used for RNA *in vitro* transcription were constructed as previously described (19). The PBS-segment mutant template plasmids for RNA transcription were generated by site-directed mutagenesis. The vector pNL4–3-CMV-EGFP used for infectivity measurement had deletion of *vif, vpr, vpu, nef* and *env* open reading frames (ORF), and contained a GFP ORF driven by a CMV promoter (26). The PBS mutations in pNL4–3-CMV-EGFP for infectivity assays were generated by multiple DNA fragment assembly using primers listed in Supplementary Tables S1 and S2. (GenBuilder Cloning Kit, GenScript). All of the sequences were confirmed by Sanger sequencing (DNA Core, University of Missouri).

### RNA in vitro transcription

All of the RNA constructs, including 5′UTR, 5′UTR^ΔTAR-PolyA^, 5′UTR^ΔPBS^, TAR-PolyA, PBS-segment and mutants, TLE, PAS stem, hairpin-control and tRNA^Lys3^, were made by *in vitro* T7 transcription reactions. The DNA templates of 5′UTR, 5′UTR^ΔTAR-PolyA^, 5′UTR^ΔPBS^, TAR-PolyA, PBS-segment and mutants were made by PCR amplifying corresponding region of the plasmids with a 5′- Top17 sequence (5′-GAAATTAATACGACTCACTATA-3′, the five extra base pairs at the 5′-end were added to promote T7 binding and enhance RNA transcription yield). The template DNAs of TLE, PAS stem, hairpin-control and tRNA^Lys3^ were custom synthesized (IDT). The nucleotide specific ^2^H labeled samples, including A^2R^GU^R^-, A^2R^C^R^U-, A^2R^C^R^U^R^-, A^2R^G^R^-, AC- and AG-PBS-segment (nomenclature here: the letters denote the nucleosides containing ^1^H which is visible by NMR, R = ribose, A^2R^ means H2 and ribose hydrogens are protonated; A^2R^GU^R^-PBS-segment means the H2 and ribose of adenosine, ribose of uracil and guanosine are protonated, and cytidine is fully deuterated) were prepared by incorporating corresponding deuterated and protonated rNTPs in T7 transcriptions as previously described (19,27). Fully deuterated rNTPs and H5,H6-deuterated CTP and UTP were purchased from Silantes (Silantes GmbH, Munich) and Cambridge Isotope Laboratories (CIL, Andover, MA). H8-deuterated ATP and GTP were prepared in the lab (19). ^13^C/^15^N labeled RNAs were synthesized by incorporating ^13^C/^15^N labeled rNTPs (CIL, Andover, MA) in T7 transcription.

### RNA refolding

The PBS-segment RNA used for biophysical studies was refolded by preparing the RNA in 10 mM Tris–HCl, pH 7.5, incubating at 95°C for 3 min, snap cooling on ice, and then mixing with salts to reach final salt concentrations of binding buffer A (10 mM Tris–HCl, pH 7.5, 140 mM KCl, 10 mM KCl and 1 mM MgCl_2_). The RNA samples were then incubated at 37°C for 30 min prior to EMSA and ITC experiments.

### Protein purification

The plasmid for recombinant RHA expression was a kind gift from Dr William Clay Brown (University of Michigan). The recombinant RHA contained N-terminal His_6_- and Mocr-tags and was expressed in insect cells. Purification of RHA was performed as previously described (20) and the protein was stored in storage buffer (10 mM Tris–HCl, pH 7.5, 140 mM KCl, 10 mM NaCl, 1 mM MgCl_2_, 1 mM β-mercaptoethanol and 10% glycerol). The recombinant N-terminally His_6_-tagged dsRBD1 was expressed in *Escherichia coli* BL21(DE3)-pLysS cells (Invitrogen) treated with 0.3 mM IPTG for 4 h at 37°C. To express ^15^N labeled dsRBD1, the bacteria were grown in the LeMaster and Richards minimal medium with ^15^NH_4_Cl (0.5 g/l) as the sole nitrogen source. The harvested cells were resuspended in lysis buffer (40 mM NaH_2_PO_4_, 300 mM NaCl, pH 7.5) with lysozyme, sonicated and centrifuged. The supernatant was applied to a Cobalt column (HisPur Cobalt Resin, Thermo Scientific), and the protein was eluted with lysis buffer containing additional 150 mM imidazole. To remove the His_6_-tag, the protein was digested overnight at 4°C with TEV protease (1 mg protease per 20 mg of His_6_-dsRBD1) and dialyzed in low-salt buffer (30 mM Tris, 100 mM NaCl, 2 mM EDTA, 5 mM β-mercaptoethanol). The protein was then subjected to ion exchange and size exclusion chromatography, and stored in storage buffer at –80°C. The plasmid for recombinant NC (HIV-1 NL4–3 strain) expression was a kind gift from Dr Michael F. Summers (University of Maryland, Baltimore County). The NC protein was expressed and purified as described elsewhere (28). The pRT-Dual plasmid (29) for recombinant RT expression (HIV-1 HXB2 strain) was a kind gift from Dr. Donald Burke (University of Missouri, Columbia). The RT protein was expressed and purified as described previously (30).

### Cells and viruses

293FT cells (Invitrogen) were propagated in Dulbecco's modified Eagle's medium (DMEM, Sigma) supplemented with 10% fetal bovine serum (FBS). Wildtype and mutant viruses were produced by transfection as previously described (26). Briefly, 293FT cells were plated in 10% FBS DMEM at density of 5 × 10^5^ cells per well in six-well plates overnight and then co-transfected with 500 ng of pNL4–3-CMV-EGFP or mutants together with 100ng pMD-G (AIDS Reagent Program), which encodes the vesicular stomatitis virus glycoprotein, VSV-G, for pseudotyping. The mixtures were added dropwise to the cells and incubated for 6 hrs in 5% CO_2_ at 37 °C, rinsed, and cultured in 2 ml of 10% FBS DMEM. The supernatant was harvested 48 h post-transfection by centrifugation at 1000 rpm for 10 min at 4°C, and the vector virus preparations passed though 0.45 μm filters. The transfected cells were collected and fixed with 4% of paraformaldehyde, and transfection efficiency was monitored by detection of EGFP on an Accuri C6 Flow Cytometer (BD Biosciences, San Jose, CA, USA). The Gag p24 was quantified by ELISA using pre-coated MicroFluere plates (Optofluidic Bioassay, MI).

### Infectivity assay

Equivalent amounts of VSV-G-pseudotyped vectors viruses (250 ng of Gag p24) with or without mutations in the PBS-segment were used to transduce 2 × 10^5^ of TZM-bl cells in 12-well plates. The infected cells were collected and fixed with 4% paraformaldehyde 24 hrs post-transduction, and the percentage of cells expressing EGPF was measured using the BD Accuri C6 Flow Cytometer (BD Biosciences, San Jose, CA, USA). The infectivity was calculated as the percentage of EGFP-expressing cells in mutant virus infected cells compared to that of the wild type (WT).

### Quantification of reverse transcription products in infected cells

MT4 lymphocytes (5×10^5^ cells in 0.5 ml of RPMI medium) were seeded in 12-well plates and incubated with equivalent cell-free medium containing vector viruses normalized to 200 ng of Gag p24 and spinoculated for 2 h, washed twice in RPMI medium and cultured for 4 h. The inactivated virus control sample was heated to 100°C for 5 min, cooled to room temperature, centrifuged at 12 000 rpm for 1 min, and treated with DNase I for 1 h. The infected cells were collected and washed twice with 1× phosphate buffered saline and DNA was extracted in the QIAamp DNA blood mini kit (Qiagen). Cellular DNA (600 ng) was evaluated by qPCR with specific primer sets that detect early and late products of HIV-1 reverse transcription (Supplementary Table S3) (31). The abundance of copies was determined relative to standard curves generated on gene-specific PCR amplicons compared to mock and heat-inactivated virus infection.

### *In vitro* tRNA annealing, primer extension and tRNA placement assays

The annealing reaction was initiated by mixing 20 pmol PBS-segment, 20 pmol tRNA^Lys3^, and 600 pmol NC (∼6 nucleotides per NC molecule) in 20 μl of buffer B (10 mM Tris–HCl, pH 7.5, 140 mM KCl, 10 mM NaCl and 5 mM MgCl_2_). The mixture was incubated at 37°C for 30 min, and the reaction was terminated by incubating with 0.5% SDS and 0.2 mg/ml Proteinase K at 37°C for 30 min. The RNA was purified by phenol-chloroform extraction and electrophoresed on 10% polyacrylamide gels.

Primer extension assays were performed with 5′-Cy3-labeled tRNA^Lys3^ annealed on PBS-segment and mutants in the presence of NC. To label tRNA^Lys3^ on the 5′-terminus, GMP-primed tRNA^Lys3^ was synthesized by mixing GMP with rNTPs in T7 transcription, and the RNA was fluorophore labeled with a Cy3-NHS ester (Lumiprobe) (20,32). The primer extension reaction was initiated by mixing 0.33 mM dNTP with a solution containing 0.1 μM template and 0.4 μM RT, incubated in 37°C for 0, 0.5, 1.0, 1.5, 2.0, 3.0 and 4.0 min, and quenched by 25 mM EDTA. The proteins were removed from the reactions by phenol–chloroform extraction, and the primer extension products were resolved on 10% denaturing polyacrylamide gels.

To check the tRNA placement on the WT and mutant viral RNAs, 200 μl of WT and mutant pseudotyped viruses were harvest from transfected 293FT cells and treated with Dnase I and DpnI at 37°C for 90 min. The viral RNA was extracted with TRIzol (Invitrogen). To check if the viral RNA was pre-annealed with tRNA^Lys3^ to prime reverse transcription, 2 ng of extracted RNA was mixed with dNTP (10 mM each), 5× First-Strand Buffer (Invitrogen), 0.1 M DTT and RNaseOUT™ (40 units/μl), and the mixture was incubated at 42°C for 2 min. Primer extension was initiated by adding 1 μl (200 units) of SuperScript™ II RT (Invitrogen), and the reaction was incubated at 42°C for 50 min. To quantify the (-)cDNA products, 2 μl of cDNA generated from the previous step was mixed with the primer/probe set targeting the RU5 region (Supplementary Table S3) for qPCR (Luna^®^ Universal Probe qPCR Master Mix, NEB). To quantify the total gRNA, the primer/probe set (Supplementary Table S3) targeting gag region was used in RT-qPCR (Luna^®^ Universal Probe One-Step RT-qPCR, NEB). To detect if plasmid DNA were carried over through RNA extraction, the same amount of RNA (0.2 ng) and primer/probe set targeting the gag region were used in qPCR. Both qPCR and RT-qPCR were performed on CFX96 Real-Time PCR Detection System (Bio-Rad).

### NMR spectroscopy

The PBS-segment and its control fragment samples were prepared (200–300 μM) and pre-incubated in 10 mM Tris-d_11_, pH 7.5 and 1 mM MgCl_2_ at 37°C for 30 min. Two-dimensional ^1^H–^1^H NOESY and ^1^H–^1^H TOCSY data for control RNA fragments, and ^1^H–^1^H NOESY data for fully protonated and site-specific deuterated PBS-segment RNA and in complex with dsRBD1 were collected in D_2_O (99.96%, CIL) at 308K. ^1^H–^13^C HMQC data were collected for ^13^C/^15^N labeled control RNA fragments. One- and two-dimensional imino proton spectra were collected for the PBS-segment RNA and control RNAs (200–300 μM) prepared in buffer containing 10% D_2_O + 90% H_2_O. HNNCOSY data were collected for ^13^C/^15^N labeled TLE stem loop RNA. ^1^H–^15^N TROSY data were collected for 200 μM dsRBD1 titrated with 0, 54, 108, 136 and 160 μM of the PBS-segment RNA in binding buffer B (10 mM Tris-d_11_, pH 7.5, 500 mM KCl and 1 mM MgCl_2_) in 10% D_2_O and 90% H_2_O at 318 K. All the NMR data were collected on a Bruker Avance III 800 MHz spectrometer equipped with TCI cryoprobe (NMR Core, University of Missouri). The NMR data were processed by NMRPipe (33) and analyzed by NMRViewJ (34).

### SAXS data collection and processing

SAXS data of the PBS-segment RNA were collected at Sector 18 of the Advanced Photon Source, Argonne National Laboratory. In-line data were collected by eluting 9 mg/ml PBS-segment on a Superdex-200-Increase SEC column (GE) at 0.75 ml/min flowing rate. SAXS data of the mutant RNA samples were collected at beamline 12-ID-B of Advanced Photon Source at Argonne National Laboratory, using an in-line AKTA micro FPLC setup with a Superdex 75 Increase 5/150 GL size exclusion column. The wavelength, λ, of X-ray radiation was set to 0.9322 Å. Scattered X-ray intensities were measured using a Pilatus 2M detector. The sample-to-detector distance was set such that the detecting range of momentum transfer *q* [ = 4π sin θ/λ, where 2θ is the scattering angle] was 0.005–0.85 Å^–1^. The sample passed through the FPLC column and was loaded to a flow cell for SAXS measurements. The flow cell is a cylindrical quartz capillary 1.5 mm in diameter and 10 μm wall thickness. The exposure time was set to 1 s to reduce radiation damage and data were collected at every other second. The 2D scattering images were converted to 1D SAXS (*I*(*q*) versus *q*) curves through azimuthally averaging after solid angle correction and then normalizing with the intensity of the transmitted X-ray beam flux, using the beamline software. The SAXS data were then processed using ATSAS suite 2.8 and 3.0 (35). The radius of gyration (*R*_g_) was determined by the Guinier plot in its linearity region and pairwise distribution was plotted using GNOM (36). The *ab initio* models were generated by averaging 10 independent DAMMIF runs on DAMAVER (37).

### Structure calculation

The PBS-segment structure was initially calculated using NMR-derived restraints by CYANA (38). Standard torsion angle restraints were used for regions of A-helical geometry in PAS stem and TLE stem loop. Standard hydrogen bonding restraints for Watson–Crick pairs in PAS stem (nt 123–131 and 217–225), TLE stem loop (nt 135–150, 158–167 and 171–177), and tRNA annealing arm (nt 187–189 and 195–197), and phosphate distances in A-form RNA helical structures in these regions were employed to maintain the major groove width in an A-form helical structure (Table 1) (38,39). The theoretical solution scattering curves of the lowest structures were calculated and compared with the experimental SAXS data by CRYSOL v2.8 (40). A total of 10 structures of lowest energy and lowest χ^2^ values were selected for further molecular dynamics (MD) refinement. For each of the 10 structures, 1 ns MD simulation was performed in 1 mol/L NaCl solution under 300 K and 1 bar with NMR restraints using Amber software package (41), and the structure of lowest χ^2^ value ranging from 1.8 to 2.5 in the trajectory was extracted for further refinement. In the third step, Xplor-NIH software (42) was used to further refine the structures to reduce χ^2^ values. For each of the 10 structures, 100 simulated annealing MD simulations were performed with NMR and SAXS restraints. After Xplor-NIH refinement, the χ^2^ values of the 1000 (10 × 100) structures were reduced to 1.35–1.5. The 10 structures of the lowest energies were deposited to the RCSB protein databank (PDB: 7lva).

**Table 1.** NMR Restraints and statistics of calculated structures

Cyana^a^	
NOE-derived restraints	504
Intra-residue	184
Sequential	306
Long range |*i* – *j*| > 1	14
H-bond restraints	170
NOE restraints/residue	4.89
Torsion angles	434
Target function (Å^2^)	0.2 ± 0.008
Upper distance viol. (Å^2^)	0.0114 ± 0.0002
Lower distance viol. (Å^2^)	0.0026 ± 0.0003
Sum VDW viol. (Å^2^)	1.25 ± 0.85
RMSD (Å)	2.9 ±1.6
SAXS	
χ^2^	1.4 ± 0.02
MolProbity analysis^b^	
Clashscore	0, 100th percentile (0)
Probably wrong sugar pucker (%)	1 (0.97)
Bad backbone conformation	3 (12.6)
Bad bonds (%)	0 (0)
Bad angles (%)	2 (0.05)

^a^Statistics of 10 lowest energy models.

^b^The 10 structures refined by MD simulations were evaluated using the MolProbity Server (90,91).

### Computational mutagenesis scanning

Computational mutation scanning was performed using the Vfold2D RNA structure folding model (43–46) to predict the RNA structure with single nucleotide variation at each nucleotide position. Structure prediction of a total of 309 mutants containing single point mutation in the PBS-segment was carried out. Models of lowest energy were analyzed and grouped based on the structural changes.

### EMSA

EMSA was carried out by preparing mixture solutions of 0.5 μM RNA (5′UTR, 5′UTR^ΔTAR-PolyA^ or 5′UTR^ΔPBS^) and recombinant RHA at concentrations of 0, 0.5, 1.0, 1.5, 2.0, 2.5, 3.0, 3.5 and 4 μM in binding buffer A. To compare RHA binding to PBS-segment and TAR-PolyA, 0.5 μM RNA was incubated with 0, 1, 2, 3 and 4 μM of RHA. The mixtures were incubated at 37°C for 10 min and resolved on 1.5% agarose gels stained with 0.5 μg/ml ethidium bromide. The gels were imaged using Gel Doc XR+ (Bio-Rad). EMSA experiments were repeated three times and a representative gel is shown.

### ITC

The ITC experiments were carried out by titrating 610 μM of dsRBD1 into 17 μM PBS-segment, and titrating 1.15 mM of dsRBD1 into 18 μM hairpin-control RNA at 30°C on a VP-ITC (MicroCal, GE Healthcare). Heat of dilution titrations were performed by titrating the dsRBD1 protein into a matching buffer in the same experimental settings. Both protein and RNA samples were prepared in binding buffer B. The baseline was corrected by subtracting the heat of dilution, and the data were fitted using ‘one-site’ non-linear least square regression for hairpin-control and ‘two-site’ non-linear least square regression for PBS-segment. The statistics of thermodynamic parameters (*N*, *K*_d_, Δ*H*, Δ*S* and Δ*G*) were summarized from three experiments (Supplementary Table S5).

### Molecular docking

Molecular docking of dsRBD1 onto PBS-segment was guided by NMR experimental data. Using the crystal structure of human RHA dsRBD1 (PDB: 3vyy) and 1040 PBS-segment RNA structures that had been relaxed in MD simulations, a free docking by our in-house MDockPP program (47) was carried out to generate a total of 56 160 000 potential complex structures. The modeled complex structures were subsequently sorted by the protein–RNA interaction-specific scoring function ITScorePR in a descending order based on their energy scores (48). Then, the NMR-derived constraints related to residues of the PBS segment and the dsRBD1 were incorporated to filter out compliant candidates as described below. On the RNA side, restraints impose a maximum distance of 7 Å between both H1′ and C1 of G129 in PBS-segment and dsRBD1 because of the significantly shifted H1′ of G129 upon dsRBD1 titration, and a minimum distance of 5 Å between atom and C2 of A220 of PBS and dsRBD1 because A220-H2 remains unshifted in the NMR titration spectra. Additionally, restraints requesting U141 and U172 of PBS-segment be within 7 Å of dsRBD1 were also applied. One the protein side, restraints were generated from the ^1^H–^15^N TROSY titration data, which demand the following residues, F7, G13, K14, K16, M17, T18, Y21, K29, K54, K55 and Q58, be within 7 Å of PBS-segment. These residues exhibited significant chemical shift perturbation upon PBS-segment titration (Δδ > 0.05 ppm). Distance restraints were also set for residue Y43 that should not be within 5 Å distance of RNA because no chemical shift perturbation was observed in the TROSY titration. Any qualifying candidate must satisfy all the specified restraints, resulting in a total of 69 modeled complex structures for further evaluation with experimental data. The best model with lowest energy was further optimized with UCSF Chimera (49).

Using Modeller (50), the structure of the dsRBD2-Core (RHA without dsRBD1 and RGG domains, 149–1150) was constructed by homology modeling based on the template structure of Drosophila MLE helicase (PDB: 5aor) (51), an orthologous protein sharing 51% sequence identity with the human RHA. Molecular docking was performed using our in-house docking program, MDockPP (47). A 6^º^ angular interval was used to sample the relative orientations between the dsRBD2-Core and PBS-segment RNA. The resulting 54,000 potential binding poses were then filtered with three constraints: (i) The α1 helix of the dsRBD2 interacts with the RNA, (ii) Region 3 residues (K235, K236) interact with the RNA and (iii) The dsRBD2-Core does not have any steric clash with the dsRBD1 domain when both bind to the RNA. For constraints (i) and (ii), a 7Å distance cutoff was used to define the proximity to the RNA. Namely, the constraint (i) was met if at least one atom in a residue was within the cutoff distance to the RNA for every residue in the α1 helix. The constraint (ii) regarding K235 or K236 was likewise determined. The constraint (iii) was based on the fact that both dsRBD1 and dsRBD2-Core bind to the RNA simultaneously in physiological condition. The assessment of this constraint was based on the buried solvent accessible surface area (SASA) between the dsRBD1 and the dsRBD2-Core. The SASA was calculated using NACCESS (52). The buried SASA equals (*SASA_dsRBD_*_1_ + *SASA_dsRBD_*_2−*Core*_ − *SASA_dsRBD_*_1+*dsRBD*2*−Core*_)/2, where $SAS{A_{dsRBD1}}$ and $SAS{A_{dsRBD2 - Core}}$ stand for the SASA of dsRBD1 alone and the SASA of the dsRBD2-Core alone, respectively. $SAS{A_{dsRBD1 + dsRBD2 - Core}}$ represents the SASA of the dsRBD1 and the dsRBD2-Core when they were viewed as a single structure after the dsRBD1: RNA structure and the dsRBD2-Core:RNA structure were superimposed on the PBS-segment RNA. Due to the inaccuracy in numerical calculations and the specific algorithm of the NACCESS, it is possible to obtain a non-zero, albeit small, SASA value for two molecules that are close but not touching each other. To counter this problem, a threshold value of 30 Å^2^ was used to determine if two molecules have atomic clashes. The dsRBD1 and the dsRBD2-Core were deemed to have no atomic clash if the calculated buried SASA was below this threshold value.

## RESULTS

### The PBS-segment RNA adopts a three-way junction structure

**Figure 1.**
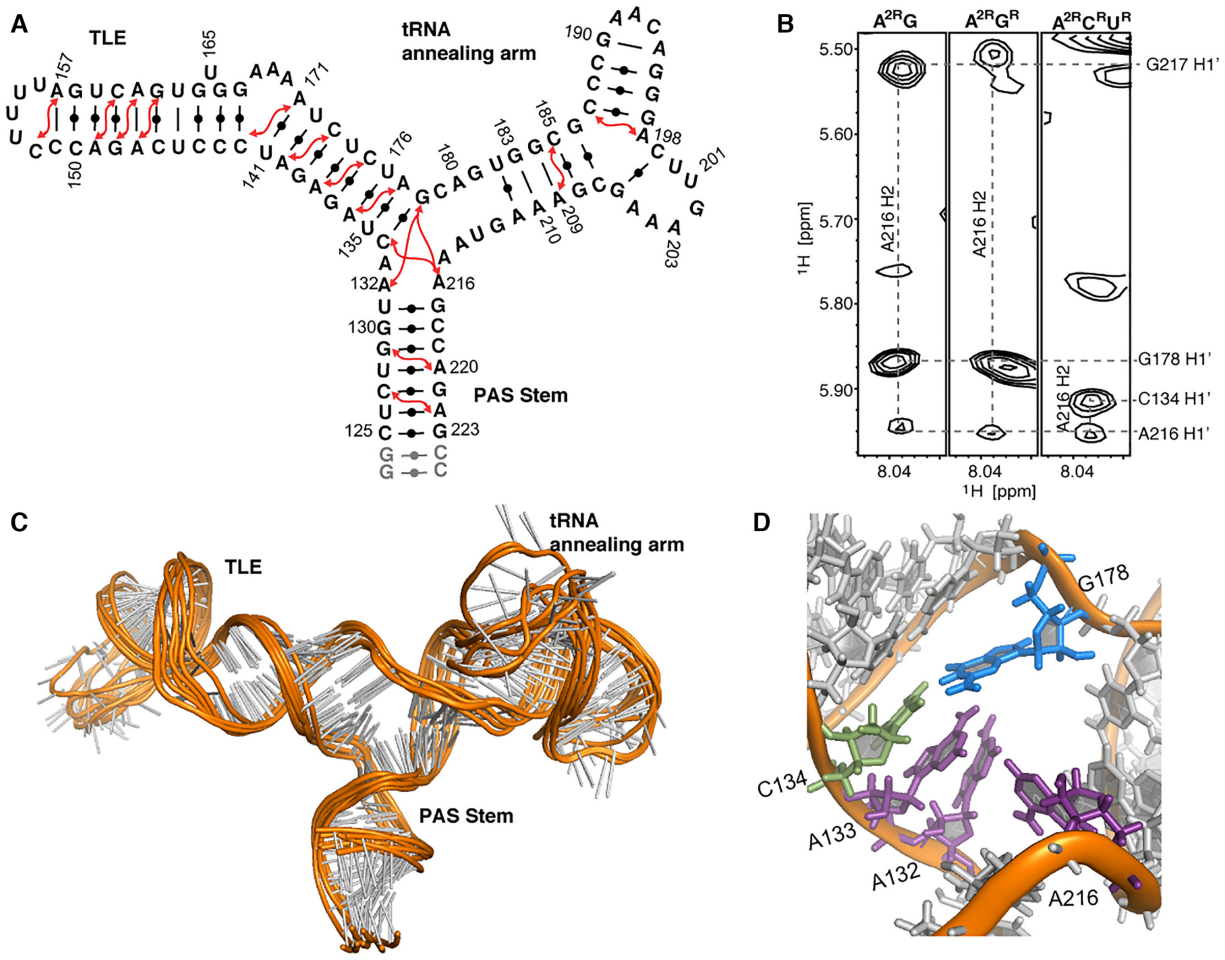
PBS-segment adopts a three-way junction structure. (**A**) Secondary structure of the PBS-segment supported by NMR assignment is shown. Red arrows denote the inter-helical NOEs involving adenosine H2 protons. (**B**) Portions of the 2D NOESY spectra with NOE connectivities supporting the three-way junction are shown. From left to right, spectra of A^2R^G-, A^2R^G^R^- and A^2R^C^R^U^R^-labeled PBS-segment. (See the nomenclature in the Materials and Methods). (**C**) NMR-derived secondary structure model of the PBS-segment. (**D**) A zoomed-in view of the adenosine-rich three-way junction in the PBS-segment.

To investigate the PBS-segment structure, an RNA of nucleotides (nts) 125–223 was synthesized with two terminal G-C pairs for efficient transcription (Figure 1A). NMR assignments were facilitated by referencing the PBS-segment RNA spectra with that of control RNA fragments, including TLE stem loop (nt 135–177) and PAS stem (nt 125–223 with a GAGA tetraloop connecting residue U131 and G217) (Supplementary Figure S1D). Attempts to make a control RNA fragment for the tRNA annealing arm were not successful. We tried various 5′- and 3′- end residues but none of the RNA fragments gave rise to similar NMR cross peak patterns in the ^1^H–^1^H NOESY spectrum as that of the PBS-segment RNA. Imino proton resonances of the PBS-segment were very broad (Supplementary Figure S1B, C). The base pairs in the PAS stem and TLE stem control RNAs were confirmed by referencing the imino proton spectrum with that of the control RNA fragment spectra (Supplementary Figure S1E, F). Additional imino proton resonances were observed and assigned to G190, G195, U200 and G206, suggesting base pairings in the tRNA annealing arm (Supplementary Figure S1C). Assignments of the non-exchangeable protons of residues in these control RNA samples were used as references for the assignment of the entire PBS-segment. To overcome the spectral overlap and enhance spectral resolution, nucleotide-specific ^2^H-labeled samples (Materials and Methods) were prepared for NMR data collection and assignments of non-exchangeable protons. This strategy enabled us to assign residues in and near the tRNA annealing arm that are not covered by the control RNA fragments. In addition to imino proton resonances, we also relied on the sequential and long-range NOEs that involve adenosine H2 to obtain secondary structure information within the PBS-segment (53) (Figure 1A). Comparison of the non-exchangeable proton assignments with database predictions also indicate the canonical structures in PAS and TLE region, and non-canonical structures in the junction and tRNA annealing arm are formed (Supplementary Figure S3) (54).

In agreement with previous structural models, our NMR data confirm the formation of PAS stem and the TLE stem in the PBS-segment (21–25). The assignment of residues in the tRNA annealing arm indicate that these residues are not completely flexible and unstructured, but instead forming some non-canonical structures. While no imino proton resonances were observed for residues U182-C185 and G208-G212, sequential stacking of these residues are indicated by the NOESY walk (Supplementary Figure S2). Long-distance NOEs were detected and assigned to the residues in the tRNA annealing arm (nt 179–214), including A209.H2–C185.H1′ (Supplementary Figure S2), and A198.H2–187.H1′, demonstrating base pairing in the tRNA annealing arm. However, some cross-peaks, such as G183.H8–U182.H1′ and A209.H2–C185.H1′, are very weak (Supplementary Figure S2), suggesting structural dynamics of these residues in the tRNA annealing arm. They are likely in rapid exchange between a stacked/paired position and unstructured positions. The junction that converges PAS stem, TLE hairpin and tRNA annealing arm is adenosine-rich (A132, A133 and A216), and NOE cross peaks between A216-H2 and the H1′s of G178, G217 and C134 were detected in spectra with different ^2^H labeling strategies (Figure 1B). In summary, our NMR data reveal that the PBS-segment RNA adopts a three-way structure with an adenosine-rich junction (Figure 1C and D).

**Figure 2.**
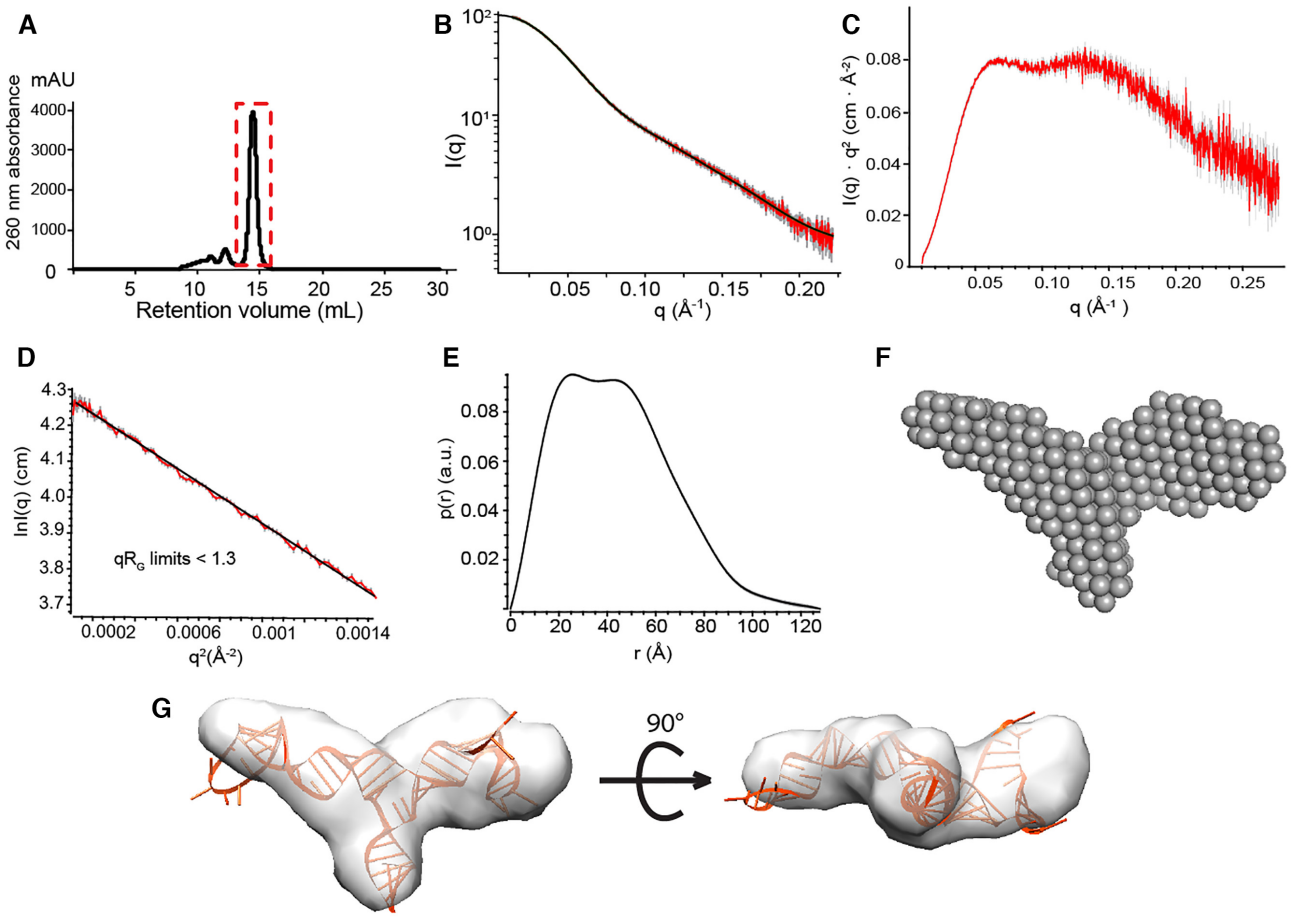
SAXS data of the PBS-segment support a three-way junction structure. (**A**) SEC profile of the PBS-segment RNA is shown. The SAXS diffraction data of red dash boxed region were used for structural analysis of the PBS-segment. (**B–E**) Averaging scattering profile (B), Kratky plot (C), Guinier plot (D) and the pair distance distribution function (E) for the scattering curve are shown. (**F**) An *ab initio* model of the PBS-segment generated with DAMMIF is shown. (**G**) Different views of the *ab initio* model superimposed with a representative structure of PBS-segment calculated by NMR-derived restraints and refined with the SAXS data.

SAXS data were collected to obtain restraints of the overall shape of the PBS-segment RNA. Gel-filtration analysis of the PBS-segment RNA showed a minor peak before the main elution peak, indicating that a small portion of the sample may be aggregated (Figure 2A). The aggregated RNAs are not likely to give rise to measurable NMR signals due to their large molecular sizes and low population (<10%), but could introduce large errors when measuring the RNA dimensions by SAXS. To obtain better homogeneity of the RNA sample, in-line SAXS data were collected by eluting the PBS-segment RNA on a Superdex-200-Increase SEC column (GE), and SAXS data collected for samples eluted in the main peak were used for structural analysis. The averaged scattering data, Kratky Plot, Guinier plot and the distribution of the interatomic distance P(r) are shown in Figure 2B–E, respectively. *Ab initio* models generated from the SAXS data recapitulated a three-way junction structure highly consistent with our NMR model (Figure 2F). Efforts were made to truncate one of the arms in the RNA sample to help unambiguously identify the TLE and the tRNA annealing arm in the SAXS *ab initio* model. However, NMR analysis of the truncated PBS-segment revealed that the secondary structure was altered due to the truncation. SAXS profiles of the NMR-derived structures were back calculated and compared with the experimental SAXS data. The structures with lowest energy and χ^2^ values were selected for further energy optimization by MD simulation (Figure 2G). The statistics of calculated structures are summarized in Table 1 and Supplementary Table S4.

### The base pairs within the three-way junction structure are phylogenetically conserved

**Figure 3.**
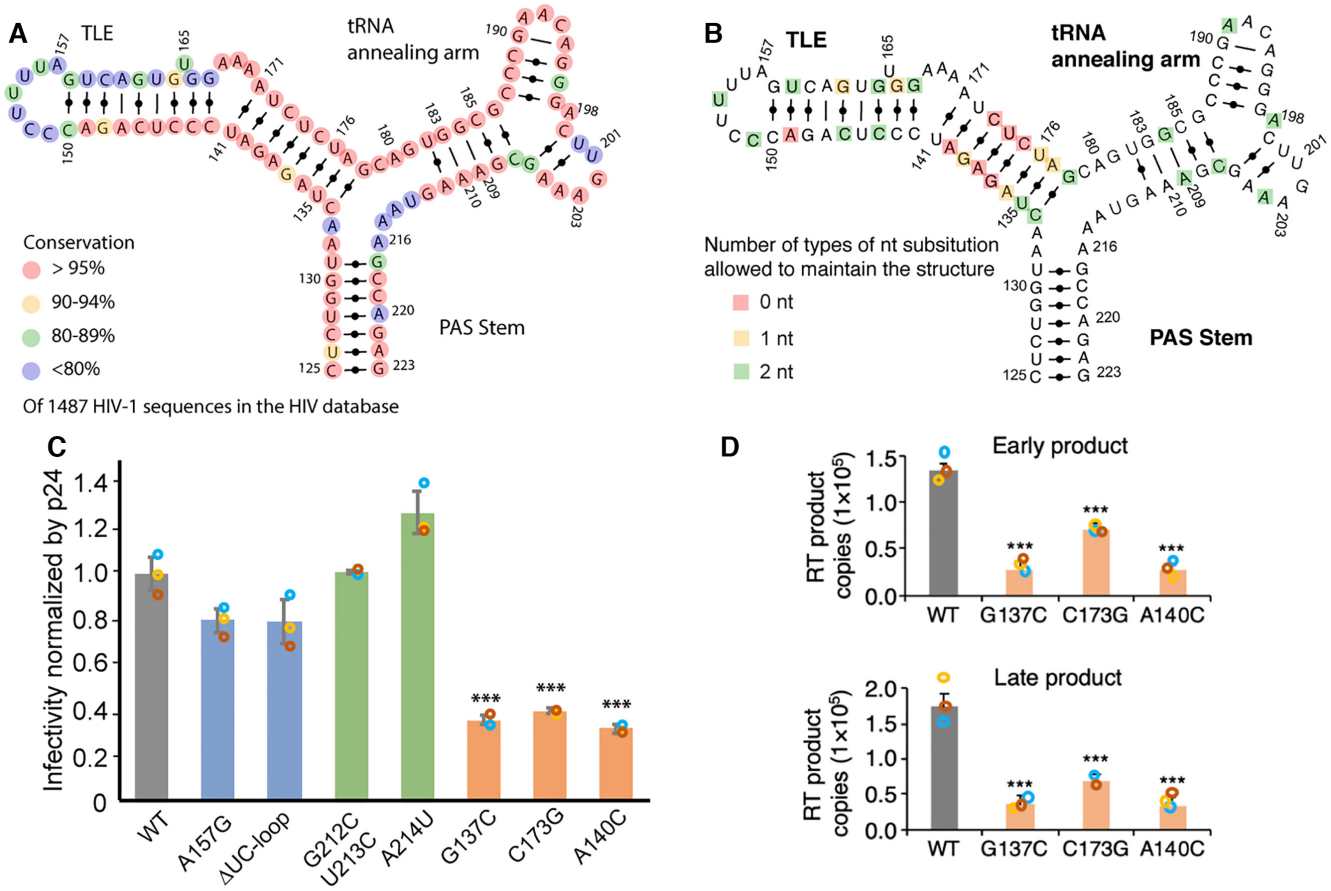
The three-way junction structure of the PBS-segment is conserved for infectivity. (**A**) Phylogenetic analysis of 1487 clinical sequences spanning the PBS-segment is shown. The conservation at each nucleotide position is color labeled. (**B**) Mutational scanning was performed to substitute each one nucleotide and predict the secondary structure of each mutant. The red, yellow, and green squares highlight the residues can be substituted by 0, 1 and 2 nucleotides in order to maintain the three-way junction structure. Unlabeled residues do not contribute to the three-way junction structure in a sequence specific manner. The low stem of TLE is shown in blue dashed box. (**C**) Mutations that disrupt the three-way structure of the PBS-segment reduced the virus infectivity. Infectivity was normalized by the p24 protein level. Gray bar, WT; blue bars, mutations in the TLE loop; green bars, mutations in the tRNA annealing arm; orange bar, mutations in the TLE stem. (**D**) Mutations that disrupt the three-way junction structure of the PBS-segment diminished HIV early reverse-transcription intermediates in target cells. Cyan, red, and yellow circles represent data from three independent experiments. Statistically significant differences were measured by Student's *t* test: *** *P* ≤ 0.001.

To determine if the three-way junction structure of the PBS-segment is phylogenetic conserved, analysis of HIV-1 sequences deposited in the HIV database (http://www.hiv.lanl.gov/) was performed. A total of 1,908 PBS-segment containing sequences (C125–G223) were aligned using ClustalX2 (55). There are 421 sequences in subtypes A, G and some circulating recombinant forms containing a 23-nt insertion downstream of the PBS (56,57). For the purposes of analysis, this group of sequences were excluded as they may result in an alternative folding of the PBS-segment (30,58–60). Analysis of the remaining 1487 HIV-1 sequences from various subtypes revealed that majority of the nucleotides in the PBS-segment are highly conserved, underlining the importance of PBS-segment in virus replication. The nearly 100% conservation of the 18-nt tRNA annealing residues (U182-C199) are expected, given necessity to maintain complementary to the 3′-end of human tRNA^Lys3^ primer for reverse transcription initiation. The PAS stem and the lower stem of TLE are structurally conserved, with base-pair substitution of U128–A220 by a U–G pair in the PAS stem, and conversion of G137–C175 to a U–A pair (Figure 3A). Maintaining the base pairing in PAS is essential for the dimeric conformation of 5′UTR and efficient reverse transcription (61–63), whereas the structural conservation of the lower stem of TLE remained unclear. We therefore hypothesized that the structural integrity of TLE lower stem is necessary to maintain the overall three-way junction structure of the PBS-segment.

To address this hypothesis, computational mutation scanning was performed to scan the nucleotides in the PBS-segment and predict their impact on the RNA secondary structure. We applied the Vfold2D RNA structure folding model (14, 15) to compute the structures for a given RNA sequence. For the WT PBS-segment RNA (HIV-1 NL4–3), the Vfold2D-predicted structure agrees with the experimental result. We performed a total of 3 × 103 (*n* = 309) single nucleotide mutations, to exhaustively predict structures for each mutant, and learned that different mutations can cause different structural changes. Critical nucleotides whose mutation cause disruption of the folding were identified. Three major types of structural changes were observed: (i) in the central three-way junction region, (ii) the TLE stem loop and (iii) the tRNA-annealing stem. Some of mutant structures maintain the WT three-way junction global fold while some form an alternative elongated stem-loop structure. We found that the disruption of the central three-way junction can result in folding changes in both the TLE stem loop and the tRNA annealing arm. The mutations that lead to the disruption of the central three-way junction are distributed mostly in the lower stem of TLE (U135–A140 and U172–G178). The result shows the TLE stem is stable as long as the lower stem is maintained. Mutations in the upper stem of TLE only cause local structural variations, but do not change the overall three-junction structure. Similarly, single point mutations in PAS and the tRNA annealing arm lead to only local changes without disrupting the three-way structure of the PBS-segment. In summary, the predicted low-tolerance nucleotides reside in the lower stem of TLE, while majority of the nucleotides outside of this region can be mutated (Figure 3B). These results indicate that the lower stem of TLE is important to maintain the three-way junction of the PBS-segment structure. Other regions are sensitive to mutations, but single nucleotide mutations only cause local structural changes.

### The three-way junction structure is important for virion infectivity

Based on the computational mutation analysis, a series of mutations were designed to (i) disrupt the TLE loop, A157G and ΔUC-loop (C151CUUUUA157 was substituted by CGAGAG), (ii) perturb tRNA annealing arm, G212C/U213C and A214U and (iii) disrupt the three-way junction structure, G137C, A140C and C173G. To avoid possible disruption of tRNA annealing and reverse transcription initiation, none of the mutation sites selected are predicted to be involved in inter-molecular interactions with tRNA^Lys3^ based on previously reported tRNA^Lys3^:PBS complex models (7–9,57,62,64–66). Vfold secondary structure prediction (43) suggests that the mutant RNAs in groups 1 and 2 adopt the three-way junction structure, whereas group 3 mutants, G137C, A140C and C173G, mainly adopt a long stem loop structure without a three-way junction feature (Supplementary Figure S4). Consistently, SAXS data reveal that these three-way junction disrupting mutants have a longer paired distance (*D*_max_), and their shapes are different compared with the WT PBS-segment (Supplementary Figure S5).

We next examined the virion infectivity of these mutants in single-round infectivity assays using an NL4–3-derived reporter vector virus. The *env* gene of the reporter virus was replaced by an EGFP open reading frame and thus EGFP was produced when the cells become transduced (26). TZM-bl cells were incubated with vector virus samples containing 100 ng of Gag p24 and harvested 24 h later. The percentage of cells producing EGFP were quantified by flow cytometry to compare infectivity. Mutations altering the TLE loop (A157G and ΔUC-loop) resulted in modest reduction of the single-round infectivity (Figure 3C), consistent with the previously reported role of TLE mimicking tRNA^Lys3^ to facilitate tRNA^Lys3^ loading for reverse transcription initiation (10). Mutations in the tRNA annealing arm showed none (G212C/U213C), or slightly positive impact (A214U) on viral infectivity (Figure 3C). These mutations are predicted to maintain the three-way junction structure (Supplementary Figure S4). On the other hand, an approximate 60% infectivity reduction was observed for the three-way junction disrupted mutants (G137C, A140C and C173G) (Figure 3C).

Since the PBS-segment is the reverse transcription initiation site for (-)cDNA synthesis, we then examined the reverse transcription activity of these mutants in lymphocytes. MT4 lymphocytes (5 × 10^5^ cells in 0.5 ml of RPMI medium) were transduced with the pseudotyped virions in cell-free medium containing 200 ng of Gag p24 by spinoculation. Six hrs post-infection, cellular DNA from the transduced MT4 cells was isolated and analyzed by qPCR using primer pairs to amplify the early and late products of reverse transcription (31). The results demonstrated that the amount of early RT product, (-)ssDNA, in MT4 cells was significantly diminished for the three-way junction disrupted mutants (G137C, A140C and C173G) (*P* < 0.001) (Figure 3D). The synthesis of late RT products was similar, indicating the significant reduction in early RT activity was carried forward. These results indicate that the three-way junction disruption decreased virion infectivity by reducing the early reverse transcription activity.

To test whether the point mutations in PBS-segment affect tRNA^Lys3^ annealing or RT loading, we examined tRNA^Lys3^ annealing by EMSA, compared the *in vitro* primer extension efficiencies, and quantified the tRNA placement on viral RNA in virions. The WT and mutant RNAs were annealed with tRNA^Lys3^ in the presence of NC. None of the mutations disrupted formation of an RNA: RNA duplex, and the migration rates of the mutant complexes on a polyacrylamide gel were similar to the WT (Supplementary Figure S6A). *In vitro* primer extension assays were carried out by incubating the annealed RNA template with RT/dNTP, and monitoring the synthesis of (–)ssDNA. No difference in the primer extension efficiency between the WT and mutants was observed (Figure S6B–E). We then investigated tRNA^Lys3^ placement on WT and mutant RNAs using RNA extracted from viral particles produced from transfected 293FT cells. The RNA was mixed with an RNase H activity reduced RT (SuperScript II, Invitrogen) and dNTPs. If the mutations in the PBS-segment do not affect tRNA^Lys3^ placement on gRNA, the co-purified tRNA^Lys3^ will serve as a primer to generate (–)cDNA when supplemented with RT (67). To normalize the (–)cDNA generated in WT and mutant viral RNAs, the input gRNAs were measured by RT-qPCR with primers/probe targeting the gag region (Supplementary Figure S6F). All of the mutant viral RNA successfully generated (-)cDNA at a similar level as the WT (Supplementary Figure S6G). The amount of proviral DNA in each sample was quantified by qPCR to ensure minimal DNA contamination (<1% RNA copy numbers). Collectively, our data show that the three-way junction disrupting mutations did not affect tRNA^Lys3^ annealing and reverse transcription under *in vitro* conditions. Their negative impact on viral replication is likely to happen prior to tRNA annealing.

### RHA preferentially binds to the PBS-segment of the HIV-1 5′UTR

**Figure 4.**
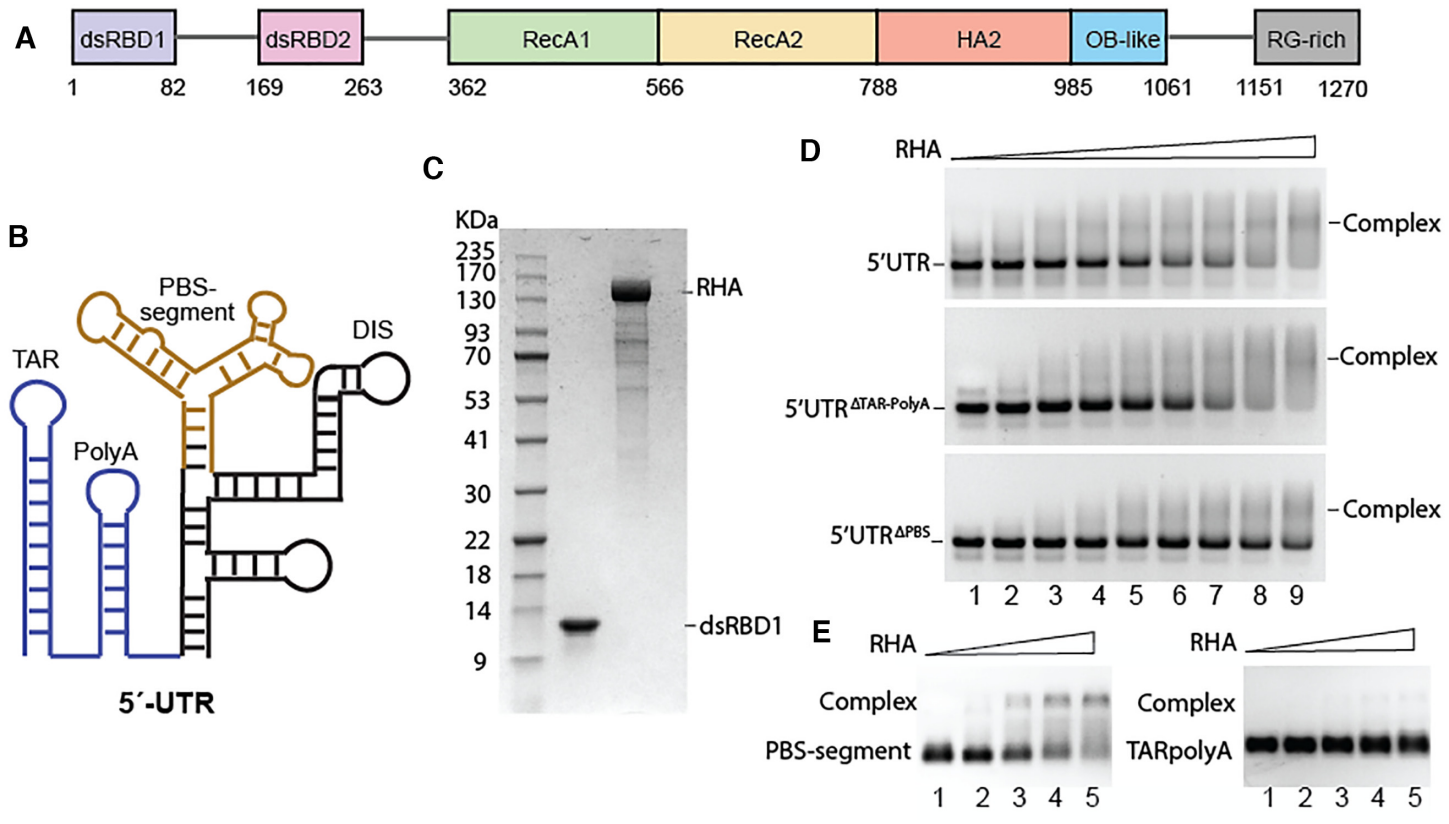
RHA preferentially binds to the PBS-segment region of the 5′UTR. (**A**) The domain organization of RHA is shown. (**B**) The secondary structural model of the 5′UTR is shown. TAR-PolyA, blue; PBS-segment, brown. (**C**) A representative SDS-PAGE shows the molecular mass markers (left), recombinant dsRBD1 purified from *E.coli* (middle), and recombinant RHA purified from insect cells (right). (**D**) EMSA of recombinant RHA binding to 5′UTR, 5′UTR^ΔTAR-PolyA^ and 5′UTR^ΔPBS^ (from top to bottom). The RNA samples were pre-dimerized, and the final mixture contained 0.5 μM of RNA and various concentrations of RHA (from lane 1 to 9, RHA concentrations were 0, 0.5, 1.0, 1.5, 2.0, 2.5, 3.0, 3.5 and 4.0 μM). The unbound RNA and complex were separated in a 1.5% native agarose gel. The representative gel of three independent experiments is shown. (**E**) EMSA of recombinant RHA binding to PBS-segment and TAR-PolyA. The final mixtures contained 0.5 μM of RNA and 0, 1, 2, 3 and 4 μM of RHA (from lane 1 to 5). The representative gel of three independent experiments is shown.

We have previously reported that HIV-1 recruits host RHA during virus assembly to serve as a processivity enhancement factor for RT (20). The recruitment is mediated by protein: RNA interactions that involve the PBS-segment of HIV-1 5′UTR (19). Therefore, the reduced infectivity and decreased reverse transcription products of the mutant viruses (Figure 3C and D) are likely due to the failure of RHA loading onto the PBS-segment. Our previous biophysical assays show that the N-terminal domain of RHA (dsRBD1 + dsRBD2) preferentially bind to the PBS-segment RNA (19). To confirm that PBS-segment is the binding target of RHA, recombinant full-length RHA protein (Figure 4A) expressed in a baculovirus system was purified (Figure 4C). The recombinant RHA was titrated to a series of HIV-1 RNA constructs, including the 5′UTR RNA (nt 1–344), 5′UTR with TAR and PolyA truncated (5′UTR^ΔTAR-PolyA^) and with PBS-segment deleted (5′UTR^ΔPBS^). These 5′UTR constructs have the 3′-half of the AUG hairpin truncated to favor RNA dimerization (Figure 4B) (53,63,68). As expected, the EMSA results show that the relative affinity of RHA for 5′UTR^ΔTAR-PolyA^ is similar to 5′-UTR, but much weaker for 5′UTR^ΔPBS^ (Figure 4D), as at RHA: RNA ratios of 7:1 and 8:1 (lanes 8 and 9) almost no free RNA bands were visible in the 5′UTR and 5′UTR^ΔTAR-PolyA^ samples, but significant amount of free RNA bands were observed in the 5′UTR^ΔPBS^ samples. When comparing the RHA binding affinity for smaller RNA fragments, TAR-PolyA (nt 1–104, 33 kDa) and PBS-segment (33 kDa), RHA preference towards the PBS-segment was observed (Figure 4E). Together, these data demonstrate that RHA preferentially binds to PBS-segment within the 5′-UTR.

### The binding interface between dsRBD1 and PBS-segment was mapped by NMR

**Figure 5.**
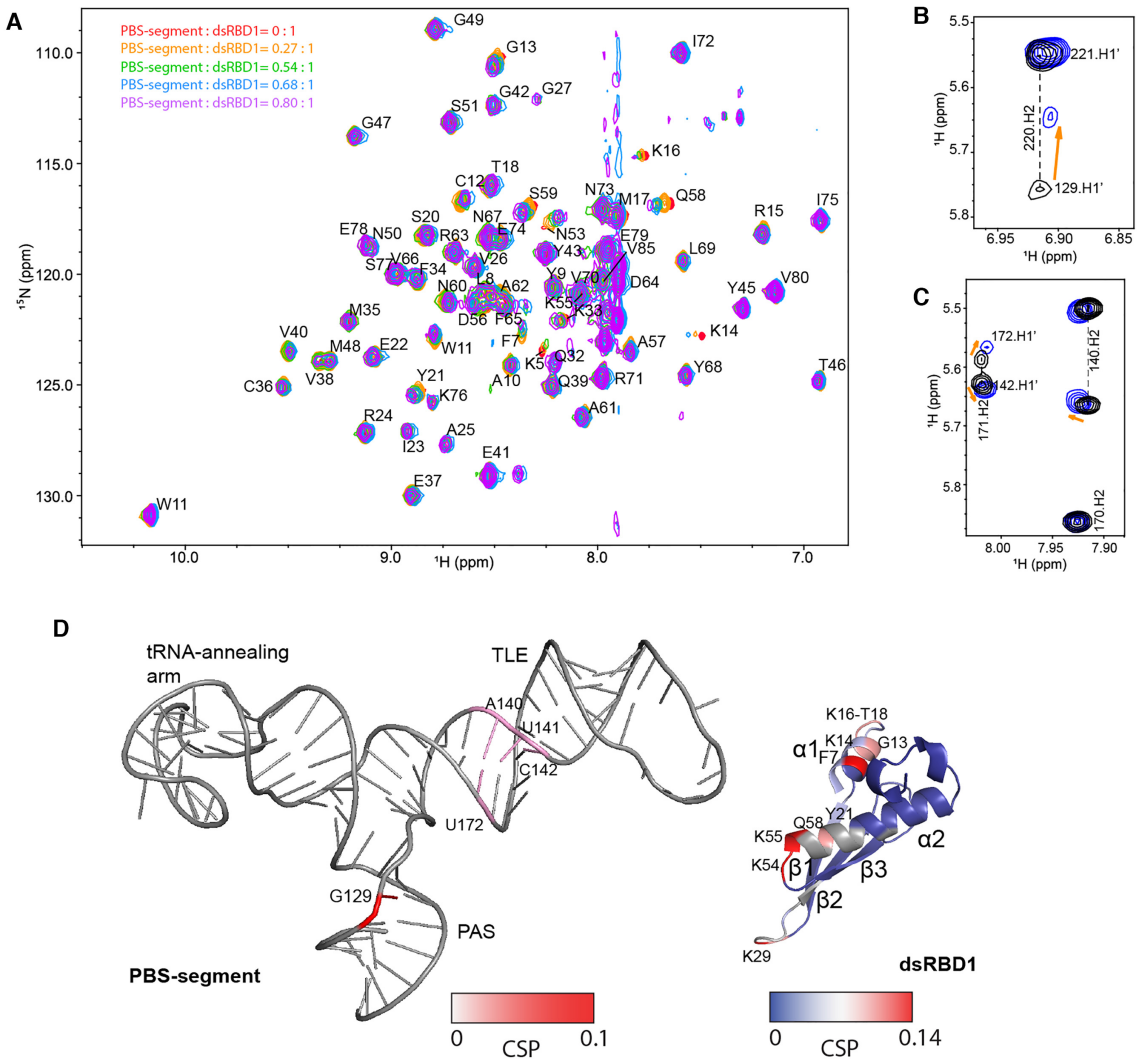
The dsRBD1:PBS-segment binding interface was mapped by NMR. (**A**) ^1^H-^15^N TROSY spectra were collected for dsRBD1 titrated with various molar equivalents of PBS-segment RNA. The RNA: protein ratios are listed on the top right corner. (**B**, **C**) Portions of the 2D NOESY spectra of the PBS-segment RNA (black) and in complex with one molar equivalent of dsRBD1 protein (blue) are shown. (**D**) Chemical shift perturbations are mapped on the structure of PBS-segment and dsRBD1.

To map the RNA-binding residues on dsRBD1, ^15^N-labeled dsRBD1 was titrated with PBS-segment in a series of ^1^H-^15^N-TROSY NMR experiments. Due to the severe precipitation issues when mixing PBS-segment RNA with dsRBD1 at NMR concentrations (200 μM), the titration was carried out in a high salt buffer and at high temperature (500 mM KCl and 1 mM MgCl_2_, 318K) to reduce non-specific electrostatic interactions between protein and RNA. As a result, the affinity between PBS-segment and dsRBD1 was weakened and thus fast exchange regime was observed (Figure 5A). Chemical shift perturbations (CSPs) were observed for a group of residues in α1 helix (region 1), the loop connecting β1–β2 (region 2) and α2 helix (region 3), suggesting these residues are within or close to the binding interface (Figure 5D and Supplementary Figure S7). These residues are consistent with the RNA binding interface reported in the crystal structure of RHA dsRBD1 in complex with a non-specific target (GC)_10_ RNA (PDB: 3vyy) (69). To identify the RNA residues within or close to the dsRBD1 binding interface, 2D ^1^H–^1^H NOESY spectra of the PBS-segment in complex with dsRBD1 was collected. In addition to fully protonated RNA, site-specific deuterated samples, including AG-PBS-segment and A^2R^C^R^U^R^-PBS-segment RNAs, were used to simplify the spectra for unambiguous assignment. G129–H1′ was shifted upon dsRBD1 titration (Figure 5B), as we previously reported (19). Chemical shifts of residues in the lower TLE stem region also exhibited moderate shifts upon dsRBD1 binding, including A140, U141, C142 and U172 (Figure 5C). The protein and RNA residues identified within and close to the binding interface by NMR titrations are labeled in Figure 5D. Both the TLE and the PAS stems are involved in dsRBD1 binding, and the distance/dimension matches the RNA binding interface in dsRBD1 (Figure 5D).

**Figure 6.**
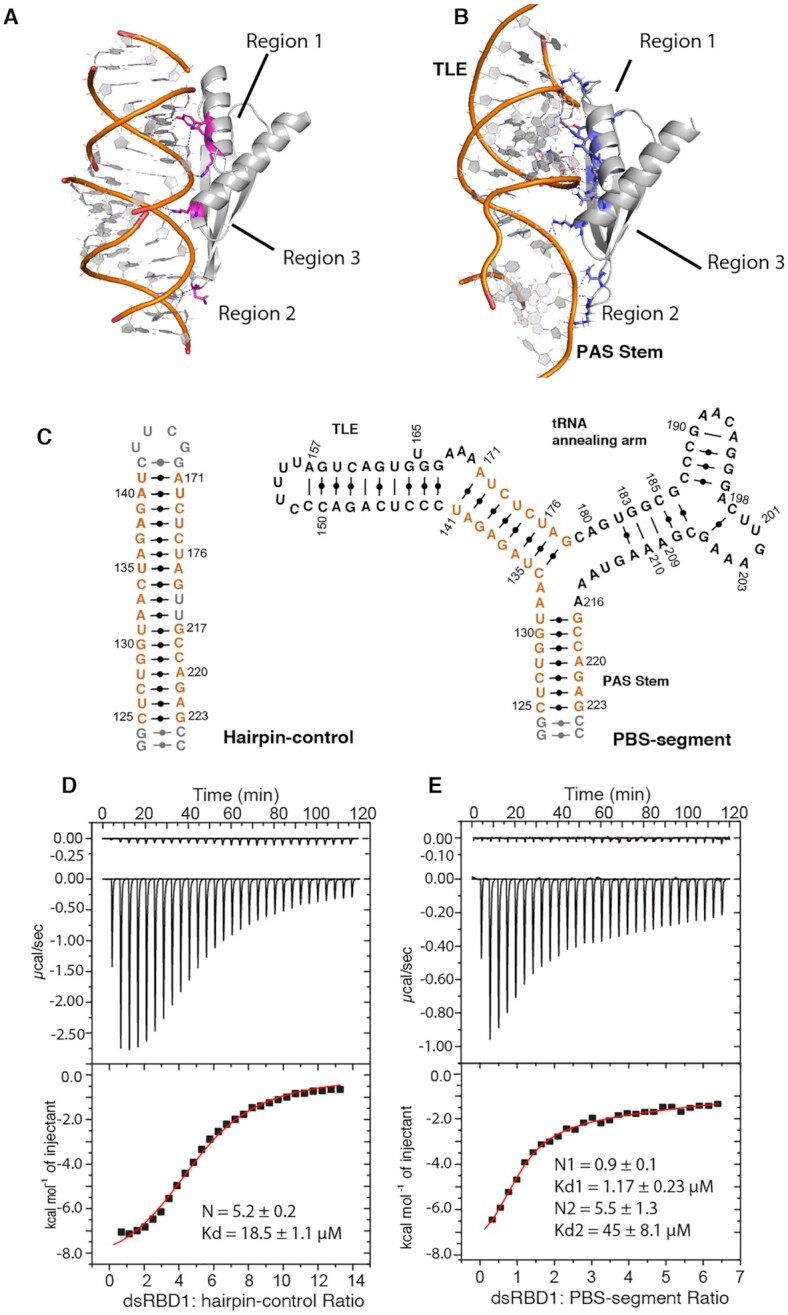
Binding of dsRBD1 to PBS-segment is of higher affinity than binding to a straight A-form dsRNA helix. (**A**) Crystal structure of dsRBD1 in complex with (GC)_10_ (PDB: 3vyy) shows that regions 1–3 interact with two consecutive minor grooves of dsRNA. The dsRBD1 residues forming hydrogen bonds with RNA are colored in magenta. (**B**) NMR data derived model of dsRBD1: PBS-segment predicts more H-bonds between protein and RNA. The dsRBD1 residues predicted to form hydrogen bonds with RNA are colored in blue. The hydrogen bonds are shown in blue dash lines. (**C**) The secondary structure of Hairpin-control (left) and PBS-segment (right) are shown. The sequence identical residues located in both RNAs are colored in orange. (**D**) ITC profile of titrating dsRBD1 into hairpin-control suggests the RNA contains several dsRBD1 binding sites. The heat of dilution profile is presented on the top panel. (**E**) ITC profile of titrating dsRBD1 into PBS-segment shows PBS-segment contains one high-affinity dsRBD1 binding site and multiple weak bind sites. The heat of dilution profile is presented on the top panel.

With the guidance of the experimental data, a data-driven molecular docking study was exploited to model the structure of the PBS-segment: dsRBD1 complex. The crystal structure of RHA dsRBD1 (PDB: 3vyy) was used to dock the PBS-segment structure using an in-house program MDockPP (47). Protein residues that are in the RNA binding interface of the reported crystal structure (PBD: 3vyy) and showed large CSP (Δδ > 0.05 ppm), including F7, G13, K14, K16, M17, T18, Y21, K29, K54, K55 and Q58, were selected to generate distance restraints (Supplementary Figure S7). The RNA residues (G129, A140, U141, C142 and U172) were also defined to be close to dsRBD1. The distance between protein and RNA was set to 7 Å because this is the shortest distance restraint that resulted in docking models without steric clashes. The lowest energy model (Figure 6B) shows that region 1 (α1 helix) of dsRBD1 interacts with the minor groove of the bottom stem of TLE, region 2 is in close proximity to the PAS stem, and K54 and K55 in region 3 are near RNA residues in or adjacent to the three-way junction (A132, A133, and U176). The TLE : regions 1 and PAS: region 2 interactions are supported by both protein and RNA NMR data (Figure 5). The NMR signals for the junction residues A132, A133, and U176 were broadened and became undetectable upon dsRBD1 titration in 2D NOESY spectra, which could be caused by protein: RNA interactions. Overall, the docking model is consistent with the NMR data, as majority of the residues near or within the protein: RNA interface exhibited CSP in the NMR titrations (Supplementary Figure S7).

The docking model suggests a shape recognition of the PBS-segment by dsRBD1. The tRNA-annealing arm introduces a ∼108° bend of the PAS away from co-axial stacking on TLE stem, resulting in ∼15 Å departure of the G129 from the straight A-form helical RNA structure model. In the crystal structure of dsRBD1: (GC)_10_ complex, in order to have all the three regions to interact with RNA, region 1 is slightly distant from the RNA so that region 2 could be close. In the case of the PBS-segment, due to the angle between the TLE lower stem and PAS, dsRBD1 is closer to RNA with regions 1–3 all in contact with RNA. This leads to formation of additional hydrogen bonds between dsRBD1 and PBS-segment as compared to a straight (GC)_10_ RNA shown in the crystal structure (Figure 6A-B).

To validate the shape-dependent recognition predicted by the docking model, ITC experiments were performed to compare the titration isotherms of dsRBD1 into PBS-segment and a hairpin-control RNA with the tRNA annealing arm truncated. The hairpin-control RNA contains base pairings in the PAS stem (nt 125–131 and 217–223) and TLE lower stem (nt 134–141 and 171–178), and is enclosed by a CG pair and a UUCG tetraloop (Figure 6C). The isothermal data of titrating dsRBD1 into the hairpin-control RNA were fit using one-site non-linear least square regression. Data fitting shows that it can interact with multiple dsRBD1 molecules (*N* = 5.2 ± 0.2) at *K*_d_ = 18.5 ± 1.1 μM, indicative of non-specific electrostatic interactions (Figure 6D). The ITC data of dsRBD1: PBS-segment titration were fit using two-sites binding mode, which shows that PBS-segment contains one high-affinity dsRBD1 binding site (*K*_d__1_ = 1.17 ± 0.23 μM) and a second set of weak binding sites (*K*_d__2_ = 45 ± 8.1 μM) (Figure 6E). The affinity of the second set of binding is comparable to the dsRBD1:hairpin-control interaction, suggesting that after the high-affinity binding site on PBS-segment is occupied, dsRBD1 starts to non-specifically interact with the RNA. It also explains that the ^1^H–^15^N TROSY NMR titrations were collected in high salt buffer and at high temperature to eliminate non-specific dsRBD1: RNA interactions (Figure 5A). Assuming no cooperativity among these dsRBD1 nonspecific binding sites, the averaged thermodynamic contribution of each dsRBD1 binding site on the hairpin-control were calculated (Supplementary Table S5). Both the PBS-segment: dsRBD1 and hairpin-control: dsRBD1 interactions are enthalpy (△*H*)-driven, but the enthalpy change associated with PBS-segment binding is significantly greater than hairpin-control binding (–7845 ± 123 cal/mol per site for the high-affinity site in the PBS-segment versus –1762 ± 37 cal/mol per site in the hairpin-control) (Supplementary Table S5). For a low affinity titration system, even though the binding event is near but not completely saturated at the end of titrations, *K*_d_ can be accurately determined but *△H* can be underestimated (70). The second set of binding sites in PBS-segment is not saturated because the heat in the final injections are not near zero, and thus the *△H* for these binding sites are less negative than the dsRBD1:hairpin-control interaction (Supplementary Table S5). In summary, the ITC data are in agreement with the docking model that more hydrogen bonds are formed when dsRBD1 binds to a specific target RNA (PBS-segment) than a non-specific target ((GC)_10_ or hairpin-control) (Figure 6A, B). Recognition of PBS-segment by dsRBD1 is RNA shape-dependent.

### Molecular docking of dsRBD2-Core onto PBS-segment suggests possible specific RHA: RNA interactions

Previous studies suggested that dsRBD2 is indispensable in directing RHA binding to target RNAs (18,69,71). Crystal structure of dsRBD2 in complex with a (GC)_10_ RNA reveals that it binds to RNA similarly as dsRBD1, with equivalent α1 helix (region 1) and loop connecting β1- β2 (region2) binding to two successive minor grooves and α2 helix (region 3) binding to the major groove in between (Supplementary Figure S8A) (69). Crystal structure of a homologous protein MLE reveals that its dsRBD2 is tightly associated with the helicase core domain. While its region 1 and region 3 are surface exposed and could interact with an RNA target, region 2 of dsRBD2 is surrounded by residues in RecA2 domain and is not surface exposed (Supplementary Figure S8B) (51). RHA and MLE share 51% sequence identity and 69% sequence similarity, so it is very likely that dsRBD2 of RHA is also in close contact with its RecA2 domain. Thus, *in vitro* study of dsRBD2 alone in RNA interactions may not represent real interactions with RNA in the context of full-length RHA, as the RecA2 domain of RHA may create spatial hindrance to prevent dsRBD2 region 2 residues from binding to a target RNA. To avoid this problem, we modeled the structure of RHA dsRBD2-Core (Figure 4A, residue 169–1150) using homology modeling based on the MLE structure (PDB: 5aor) (Supplementary Figure S8C), and docked it onto the PBS-segment RNA. The docked models were filtered using criteria that dsRBD2 regions 1 and 3 participate in RNA binding, and the dsRBD2-Core binding site does not overlap with the previously determined dsRBD1 binding site. The lowest energy model shows that the α1 helix (region 1) and K236 (region 3) of dsRBD2 interact with the three-way junction of the PBS-segment, and RecA2 residues in the Core domain make contacts with the tRNA annealing arm (Supplementary Figure S8D, E). Region 2 of dsRBD2 is not involved in PBS-segment RNA binding, as it is surrounded by residues in RecA2. These results are consistent with a previous report that substitution of a region 2 residue in dsRBD2 did not affect RHA-mediated RISC assembly (69). In summary, the docking model suggests that the dsRBD2-Core could also contribute to the recognition of the three-way junction structure of the PBS-segment.

## DISCUSSION

### The three-way junction structure of the PBS-segment is important for infectivity

Solving the structure of the PBS-segment has been challenging as the tRNA annealing arm is highly metastable thus making crystallization difficult and traditional characterization by NMR impractical. Metastable residues in the tRNA annealing arm were proposed to be unstructured by chemical probing, enzymatic probing, SAXS and computational modeling (21–25). In our study, we established the PBS-segment sequence boundaries encompassing the three-way junction in the HIV-1 RNA encapsidation signal delineated by prior NMR studies, in which residues C125-G130 form base pairs with C218–G223 (53,63). The structure reported for the PBS-segment here should resemble its parent structure in the dimeric 5′UTR prior to reverse transcription initiation that directs viral RNA genome packaging (63,72). By combining site-specific deuteration NMR strategies with SAXS methodology, EMSA with full length RHA and biological validation experiments, we were able to determine one conformation of the PBS-segment important for viral infectivity.

The identified three-way junction structure is reinforced by phylogenetic analysis of HIV sequences in patient samples, which document conserved pairings at the bottom of TLE (Figure 3A). In line with the phylogenetic findings, we show that single point mutations in this region could completely disrupt the three-way junction (Figure 3B). The mutants adopt relatively extended structures (Supplementary Figures S4–5) that failed to efficiently produce reverse transcription products in infected cells (Figure 3C, D). Our structural study of the PBS-segment implements the current knowledge of the structure of the 5′UTR of the gRNA and provide structural basis for the recruitment of beneficial host factors during genome packaging to bolster virion infectivity.

### The tRNA annealing arm is important for RHA: PBS-segment interaction

Annealing of tRNA^Lys3^ onto PBS requires a chaperone, which can be the NC domain of Gag polyprotein or the processed mature NC protein (3,4,73–75). *In vitro* primer extension on RNA extracted from protease-deficient virions exhibited lower nucleotide incorporation rate than WT, but could be rescued by incubating the RNA with mature NC protein, suggesting Gag facilitates a partial annealing of tRNA onto PBS and NC promotes a complete annealing (4). The two-step annealing model is supported by the *in virio* SHAPE analysis of the HIV-1 viral RNA in WT and protease-deficient virions (5). The matrix (MA) domain was reported to modulate the chaperone activity of Gag, which is negatively regulated by MA: RNA interactions and can be stimulated by MA interacting with inositol phosphate on the plasma membrane (76). Hence tRNA annealing promoted by Gag is likely to happen at the plasma membrane where virus assembly takes place. RHA is predominately localized in nucleus and shuttles between nucleus and cytoplasm (77). The interactions between RHA and PBS-segment may occur as early as in nucleus. It is possible that RHA is brought by the 5′UTR of gRNA to the virus assembly site, where Gag interacts with inositol phosphate and promotes tRNA annealing. The spatial and temporal control of these molecular interactions need to be investigated in future studies.

The PBS-segment structure is composed of an adenosine-rich three-way junction at the confluence of three subdomains: PAS, TLE and the tRNA annealing arm (Figure 1). It is worth mentioning that a similar SAXS envelope was reported for an almost identical PBS-segment RNA from the NL4–3 isolate (nt 125–223 with three non-native G–C pairs at the 5′/3′ termini) (24), but the residues in the tRNA annealing arm were single stranded and unstructured in the reported SAXS-derived model. In our studies, base pairings and base stackings in the tRNA annealing arm were apparent in the NMR data. Sequential NOESY walk of residues in the tRNA annealing arm was observed in the NMR spectra, but the inter-residue NOE peak intensities were weak (Supplementary Figure S2), suggesting that residues U182–C185 and G208–G212 are exchanging between flexible/unpaired and base stacked structures. The structural flexibility is believed to be favored for reverse transcription as the secondary structure must be unwound for tRNA annealing. The tRNA annealing arm in the *ab initio* model generated from SAXS data appeals wider than a typical A-form dsRNA (Figure 2G). Both the NMR and SAXS data are in agreement with an intrinsically dynamic domain in which several residues in tRNA annealing arm remain accessible for chaperone-mediated tRNA annealing.

The importance of the TLE stem loop and the PAS stem facilitating tRNA loading and activating reverse transcription have been previously addressed (7,8,10,62). Unlike PAS and TLE, residues in the tRNA annealing arm are less phylogenetically conserved (Figure 3A). Many mutations in the tRNA annealing arm are not predicted to disrupt the three-way junction structure of the PBS-segment (Figure 3B). Our study suggest that the tRNA annealing arm contributes to the overall shape of the PBS-segment RNA recognized by RHA. The bent TLE-PAS stem is preferred by dsRBD1 over the hairpin-control, a straight dsRNA helical structure which can be considered as TLE stacking on PAS (Figure 6C–E). TAR-PolyA is another example of straight helical structure as PolyA co-axially stacks on TAR (24,60). EMSA data show the affinity of dsRBD1 for TAR-PolyA is much weaker than PBS-segment (Figure 4E). Thus, even though the tRNA annealing arm does not directly interact with dsRBD1, it participates in the shape-dependent dsRBD1 recognition by preventing TLE from co-axially stacking on PAS. The affinity differences indicate two RNA-binding modes of RHA: the non-specific interactions with dsRNA when RHA functions as a helicase, and the specific interactions with PBS-segment recruited by HIV-1 as a beneficial host factor.

### RHA dsRBD1 shape-dependent recognition contributes to loading RHA to the PBS-segment

DsRBDs exist in many proteins and influence various steps of RNA metabolism. Some of the proteins have more than one dsRBD, and the contribution of each dsRBD to target RNA selection is usually not equal. MLE helicase facilitates the incorporation of the roX lncRNA into the dosage compensation complex via its dsRBDs interacting with the roX lncRNA. Studies revealed that dsRBD2 has 10-fold greater affinity than dsRBD1, and dsRBD2 plays the major role in recognition of the R2H1 and SL7 stem loops of roX lncRNA (78,79). Both the R2H1 and SL7 stem loops contain Watson-Crick and G: U wobble base pairs that form straight A-form helices. The crystal structure of dsRBD1+dsRBD2 in complex with R2H1 shows that MLE dsRBD1 binds to RNA (PDB: 5ztm) in a similar way as RHA dsRBD1 binds to a (GC)_10_ dsRNA (PDB: 3vyy). These dsRBD1: dsRNA crystal structures indicate that dsRBD1 binds to straight A-form dsRNA helices without sequence specificity.

Efforts have been made to investigate the sequence and shape recognition of dsRBDs in directing the proteins to their target RNAs. In general, dsRBDs use conserved residues in region 1, 2 and 3 to interact with two consecutive minor grooves in dsRNAs (78,80–85). Sequence specificity is achieved by some species-specific amino acids interacting with non-canonical structures in RNA. For example, dsRBD of Rnt1p, a member of RNase III family of dsRNA endonucleases, binds to the sn47 precursor RNA to direct the endonuclease to its RNA substrates by recognition of the AGNN tetraloop of the target RNAs. NMR studies show that in addition to using regions 1–3 interacting with two consecutive minor grooves in sn47 RNA, S376 and R372 in α1 form hydrogen bonds with the 2′OH of the two non-conserved 3′-nucleotides of the tetraloop, and M368 is stacked on the ribose of the adenosine in the tetraloop (86). The interactions between the dsRBM (double-stranded RNA binding motifs, an alternative name of dsRBD) of adenosine deaminases that act on RNA (ADARs) and its target GluR-2 RNA provide another example of specific recognition. ADARs are a group of enzymes that selectively deaminate adenosine. Solution studies of dsRBMs of ADAR2 in complex with GluR-2 RNA show that the dsRBM1 of ADAR2 binds to the top stem of the GluR-2 RNA with specific contacts between α1 of dsRBM1 and RNA tetraloop. The interactions include M84 making a sequence-specific contact with the A–U pair that is adjacent to the UCCG tetraloop, and E88 forming a hydrogen bond with the amino group of the first C in the tetraloop (87). UCGG belongs to the UNCG tetraloop family and adopts a specific tetraloop fold with extra thermostability (88). Therefore the recognition of Glu-R2 by ADAR2 dsRBM1 is sequence-specific and shape-dependent.

Here we show that dsRBD1 preferentially binds to the PBS-segment RNA by recognizing the TLE and PAS stems which are not co-axial stacked in the three-way junction structure. ITC data demonstrate that the dsRBD1: PBS-segment interaction is of higher affinity compared to binding to a straight A-form helical RNA structure (hairpin-control, Figure 6C–E). K5, N6, Y9, K54, K55, K29 and N30 are involved in H-bonds with RNA in both the crystal structure of dsRBD1: (GC)_10_ complex and our dsRBD1: PBS-segment model. In addition, K16, T18, Y21, K29, N23 and E41 participate in H-bonds with PBS-segment, which explains the *K*_d_ and enthalpy differences between dsRBD1 binding to a straight helical RNA and PBS-segment. Some of these residues are unique in the dsRBD family (89), and therefore they are likely to contribute to the shape-dependent recognition by dsRBD1.

In summary, we show that the structure of the PBS-segment prior to tRNA annealing folds into a three-way junction structure that is important for viral infectivity. The biophysical studies of the interactions between RHA dsRBD1 and the PBS-segment provides a plausible explanation for the recruitment of a host factor beneficial for the late stage virus replication. Although we cannot exclude the possibility of other yet-to-be discovered molecular interactions involving PBS-segment, our solution structure of the PBS-segment provides the structural basis for further investigation of the biomolecular interactions that occur on the scaffold of the PBS-segment.

## DATA AVAILABILITY

Atomic coordinates for the PBS-segment RNA structure have been deposited into the RSCB Protein Data Bank under the accession number 7LVA. Chemical shifts have been deposited to the BMRB under accession number 30868. SAXS data for PBS-segment have been deposited to the SASBDB under accession number SASDJU7.

## Supplementary Material

gkab342_Supplemental_File
