# Supplementary material for: The three-way junction structure of the HIV-1 PBS-segment binds host enzyme important for viral infectivity
Source: Nucleic Acids Res. 2021 May 12;49(10):5925–42. doi: 10.1093/nar/gkab342 (PMC8191761; doi:10.1093/nar/gkab342)
Supplement: gkab342_Supplemental_File [file gkab342_supplemental_file.pdf]

Supplementary

Tripartite structure of the HIV-1 PBS-segment binds host enzyme important for viral infectivity

Zhenwei Song[1], Thomas Gremminger[1], Gatikrushna Singh[2], Yi Cheng[1,3,4], Jun Li[1,3,4], Liming Qiu[1,3,4,5], Juan Ji[1], Margaret J. Lange[6], Xiaobing Zuo [7], Shi-Jie Chen[1,3,4], Xiaoqin Zou[1,3,4,5], Kathleen Boris-Lawrie[2*] and Xiao Heng[1]*

[1] Department of Biochemistry, University of Missouri, Columbia, MO, 65211

[2] Department of Veterinary and Biomedical Sciences, University of Minnesota, Saint Paul, MN, 55108

[3] Department of Physics and Astronomy, University of Missouri, Columbia, MO, 65211

[4] Institute for Data Science and Informatics, University of Missouri, Columbia, MO, 65211

[5] Dalton Cardiovascular Research Center, University Missouri, Columbia, MO, 65211

[6] Department of Molecular Microbiology and Immunology, University of Missouri, Columbia, MO, 65211

[7] X-Ray Science Division, Argonne National Laboratory, Lemont, IL, 60439

* Correspondence: hengx@missouri.edu and kbl@umn.edu

Keywords: HIV-1 PBS-segment, DHX9/RHA, RNA structure, NMR, SAXS, Molecular docking

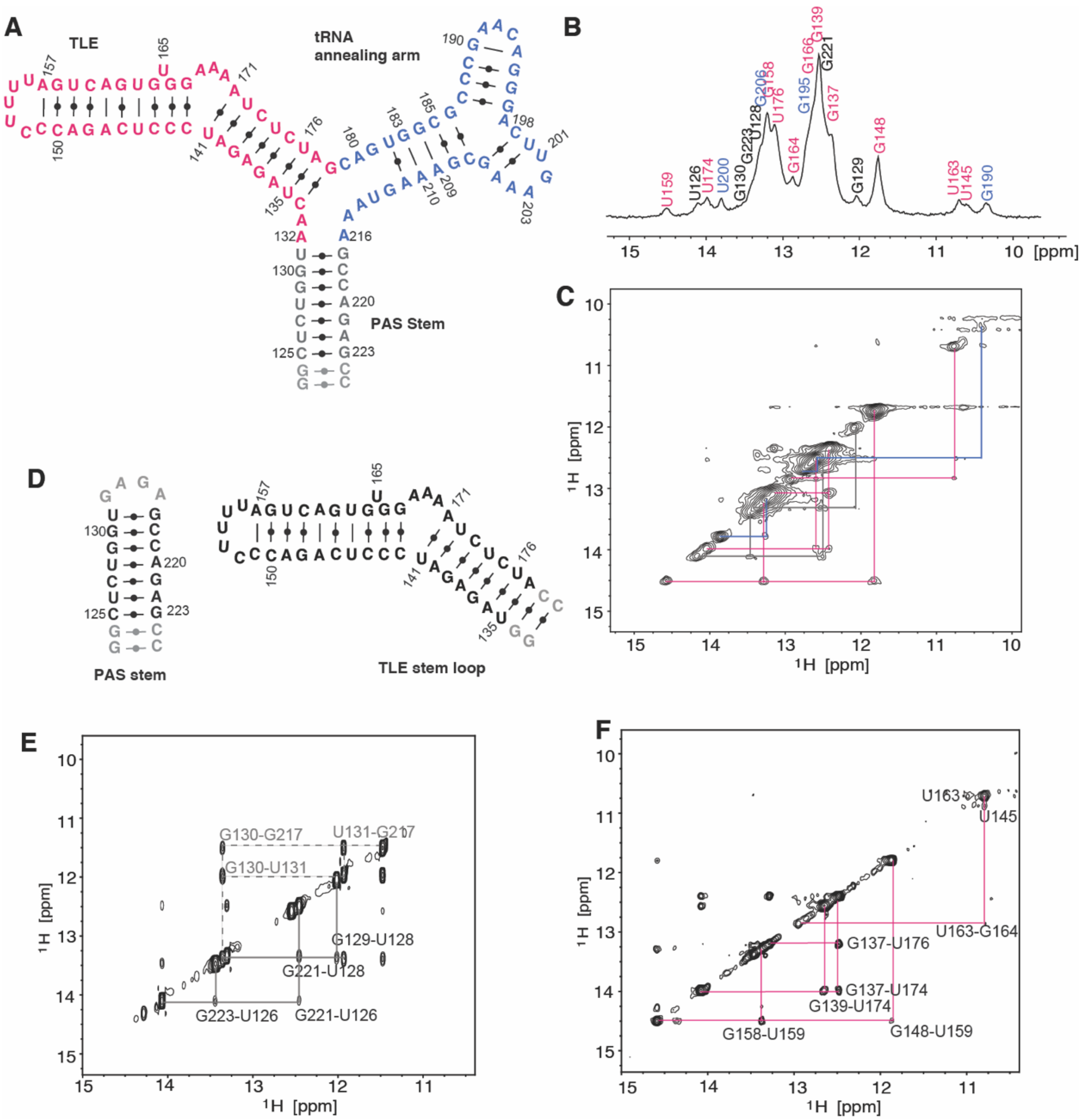

Supplementary Figure 1. Imino proton spectrum supports the formation of the TLE and PAS stem in the PBS-segment. **A**. Secondary structure of the PBS-segment. **B**. 1D imino proton spectrum of the PBS-segment. Assignment of chemical shifts from PAS stem, TLE and tRNA annealing arm are shown in gray, red and blue, respectively. **C**. ^{1}H-^{1}H NOESY spectrum for the imino-proton correlation of the PBS-segment RNA. The imino-proton correlations in PAS, TLE and tRNA annealing arm are traced in gray, red and blue respectively. **D**. Secondary structure of PAS stem and TLE stem loop RNAs to serve as controls for NMR assignment. **E-F**. ^{1}H-^{1}H NOESY spectrum for the imino-proton correlation of PAS stem (E) and TLE stem loop (F). The dashed lines denote NOE connectivities for the non-native residues in the PAS stem that do not exist in PBS-segment.

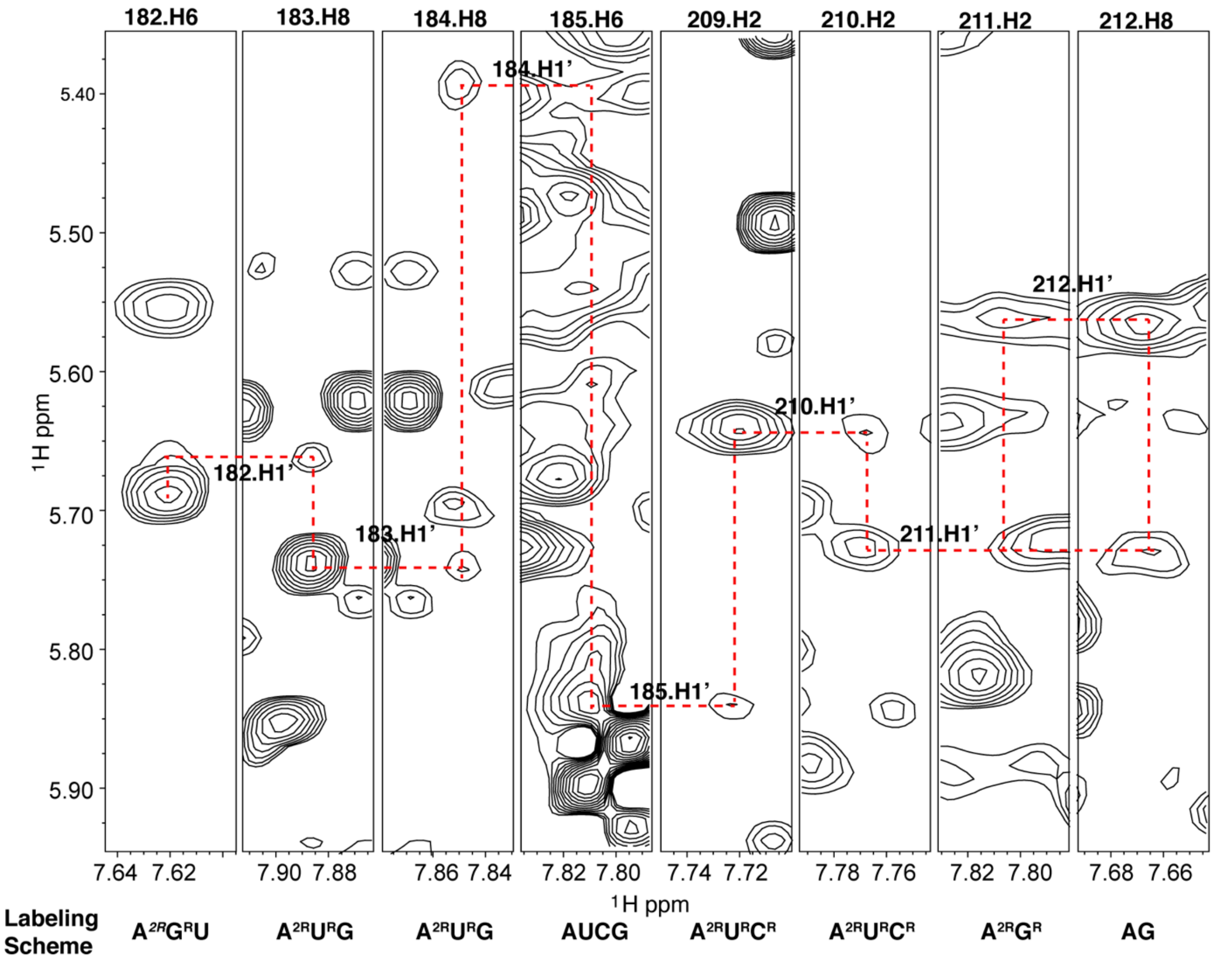

Supplementary Figure 2. Plots of ^{1}H-^{1}H NOESY spectra from PBS-segment collected with different labeling strategies. NOE connectivities of residues in the tRNA annealing arm of the PBS-segment are shown in red dashed line. Base stacking from U182 to C185 (left four strips) and from A209 to A212 (right three strips) were observed. The cross-helical NOE between A209.H2 and C185.H1’ (the 5th strip from the left) indicate the distance proximity between the two RNA chains. The RNA labeling scheme is shown on the bottom.

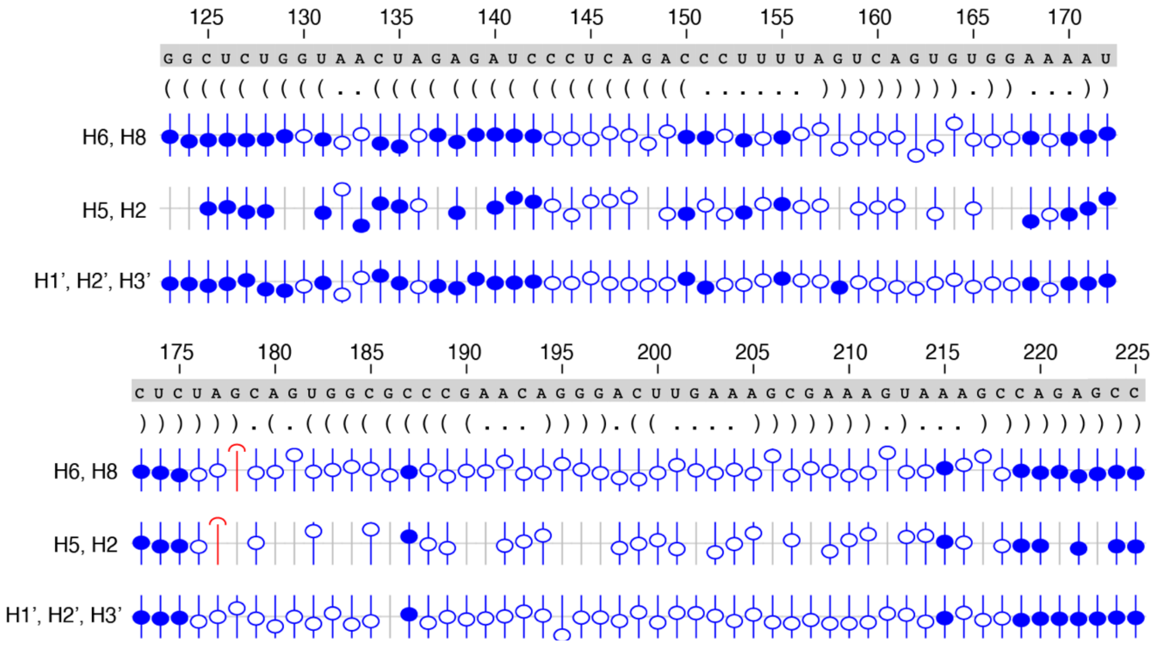

Supplementary Figure 3. The sequence, secondary structure, and NMR assignment validation for PBS-segment are shown. The secondary structure is shown in Vienna dot-bracket format. The assignments of H6/H8, H5/H2, and H1'/H2'/H3' were validated by NMRView chemical shift prediction software. Filled circles denote the assigned proton chemical shift deviation from reported chemical shifts in the BMRB database, and the open circles denote the chemical shift deviation from chemical shift predictions. Assignments that fall outside of the tolerance range (± 0.5 ppm) are indicated with a red arrow. A177 and G178 are near the three-junction and thus their chemical shifts are away from predicted positions.

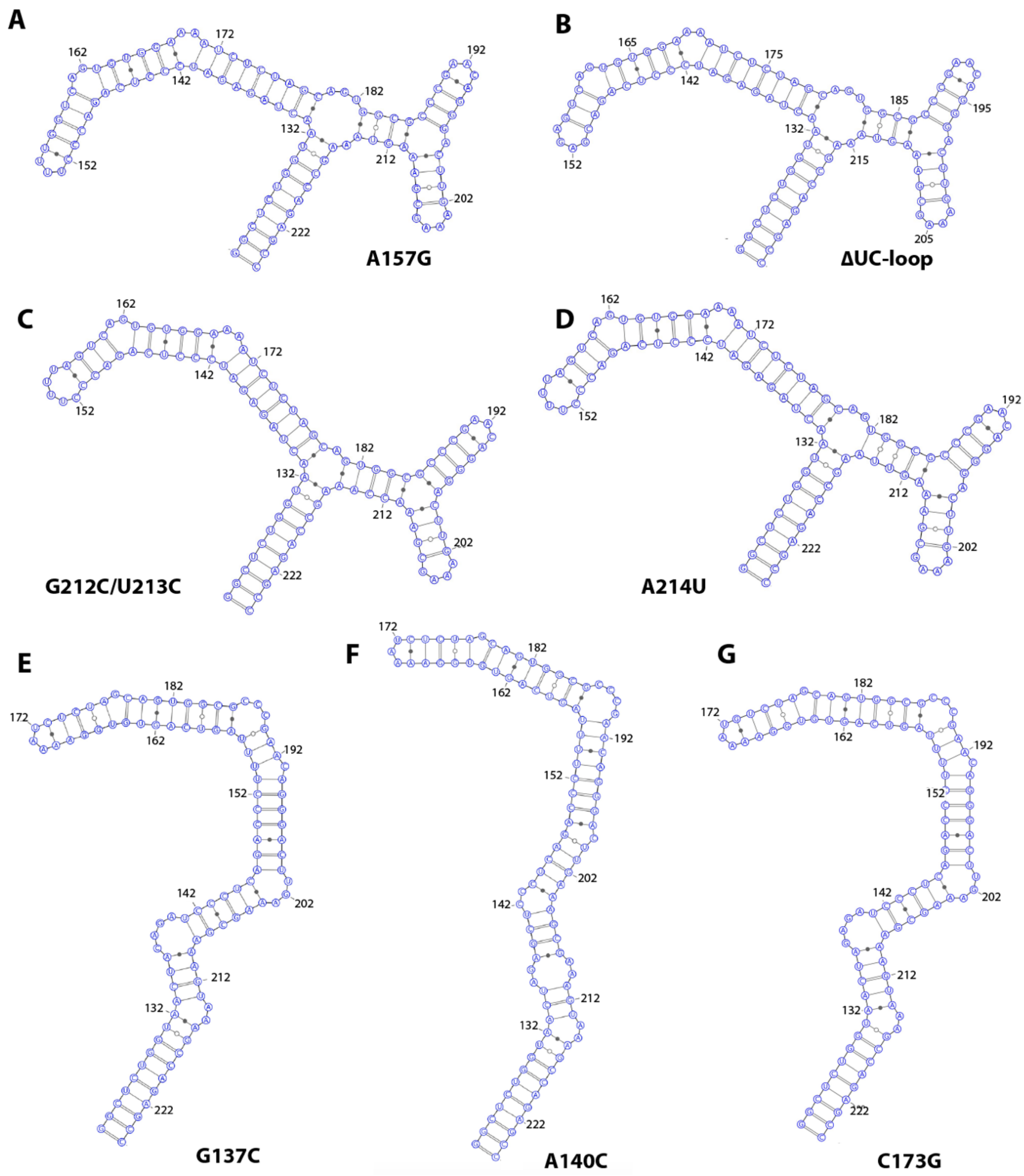

Supplementary Figure 4: Predicted secondary structures of the PBS-segment mutants that were tested in the viral infectivity assays. Mutations in the TLE loop, including **A.** A157G, **B.** ΔUC-loop, and mutations in the tRNA annealing arm, including **C**. G212C/U213C and **D.** A214U, do not disrupt the three-way junction structure. Mutations in the lower TLE stem, including **E**. G137C, **F**. A140C and **G**. C173G resulted in an extended structure. All predicted secondary structures were performed using Vfold2D RNA structure folding model.

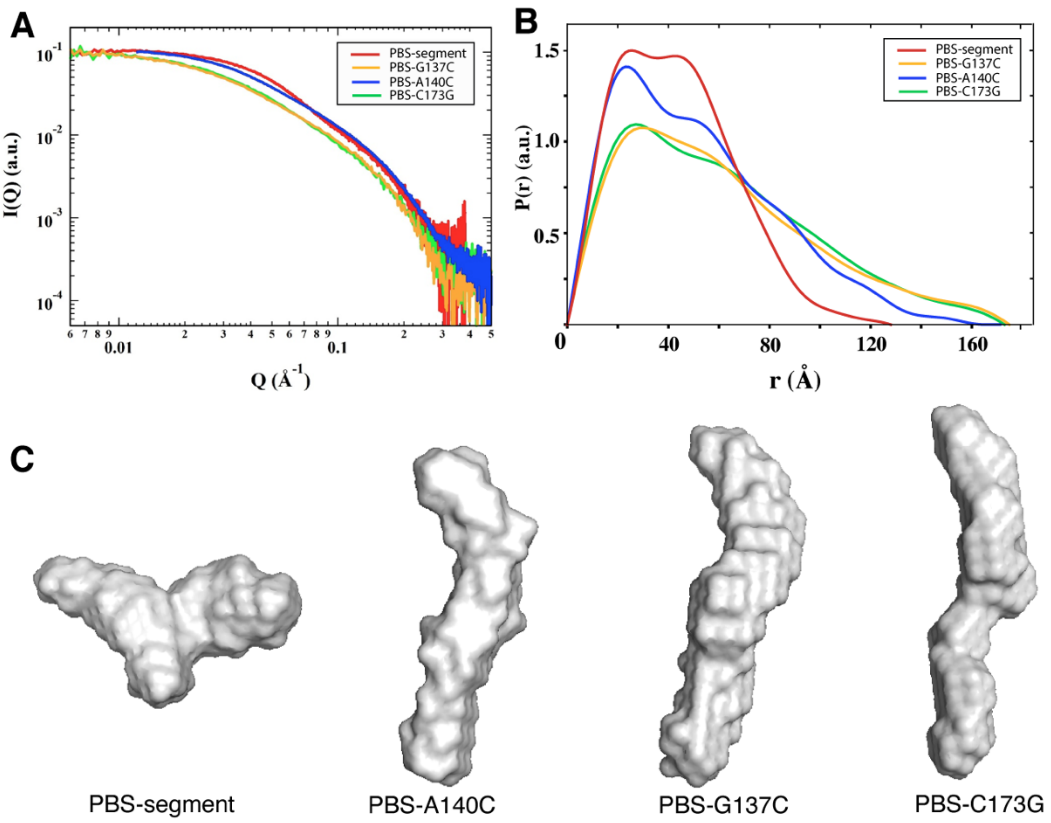

Supplementary Figure 5. SAXS data of the PBS-segment RNA and mutants show that point mutations in the lower stem of TLE altered the RNA structure. A. Overlay of averaging scattering profiles of PBS-segment, PBS-G137C, PBS-A140C and PBS-C173G is shown. **B**. Overlay of the pair distance distribution function of PBS-segment and the mutants is shown. **C**. *Ab initio* models were of PBS-segment and the mutants were generated by DAMMIF.

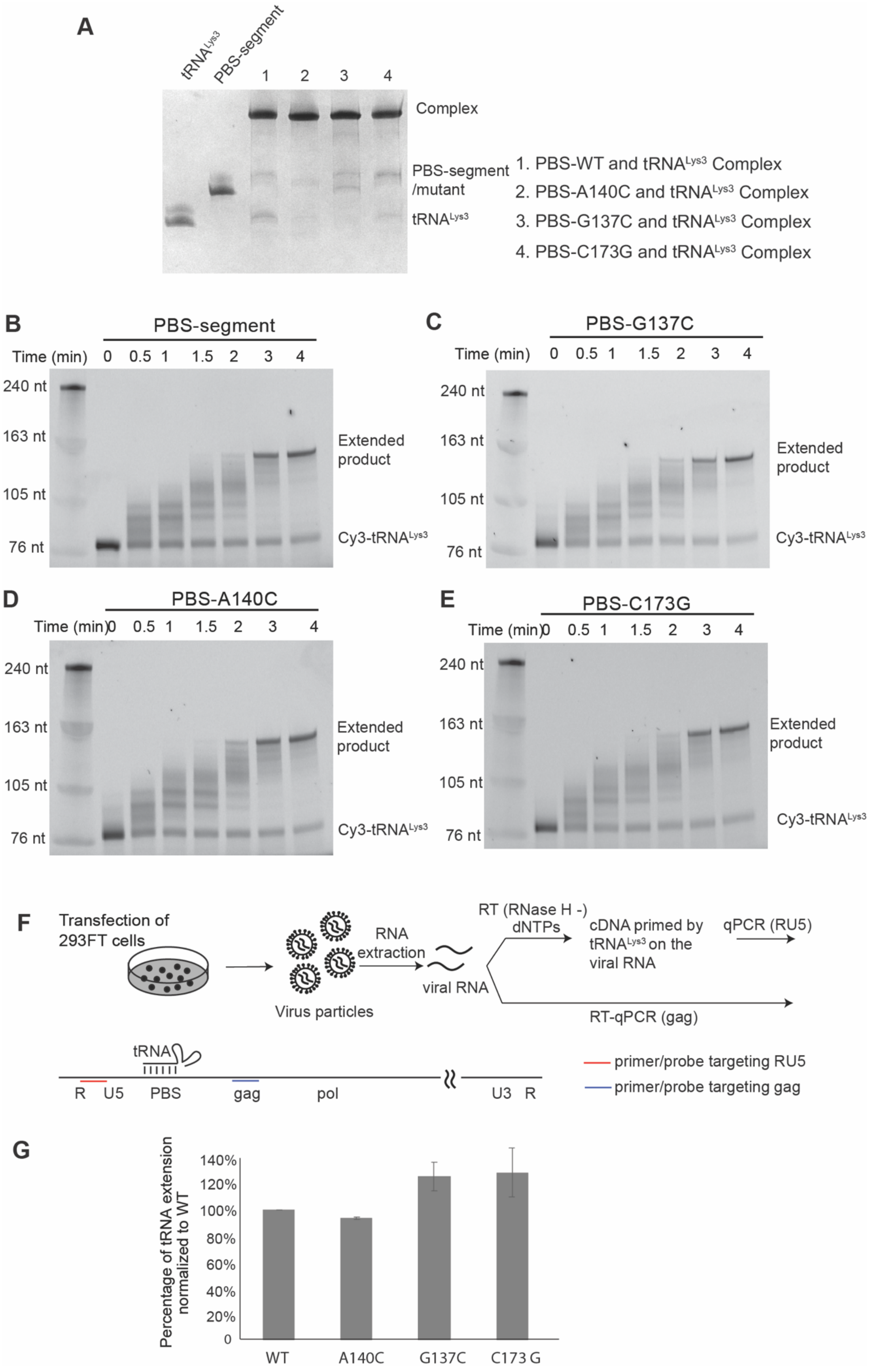
A
tRNALys3
PBS-segment
1 2 3 4
Complex
PBS-segment
/mutant
tRNALys3
1. PBS-WT and tRNALys3 Complex
2. PBS-A140C and tRNALys3 Complex
3. PBS-G137C and tRNALys3 Complex
4. PBS-C173G and tRNALys3 Complex
B
PBS-segment
Time (min) 0 0.5 1 1.5 2 3 4
240 nt
163 nt
105 nt
76 nt
Extended product
Cy3-tRNALys3
C
PBS-G137C
Time (min) 0 0.5 1 1.5 2 3 4
Extended product
Cy3-tRNALys3
D
PBS-A140C
E
PBS-C173G
F
Transfection of 293FT cells
Virus particles
RNA extraction
viral RNA
RT (RNase H -) dNTPs
cDNA primed by tRNALys3 on the viral RNA
qPCR (RU5)
RT-qPCR (gag)
tRNA
R U5 PBS gag pol U3 R
primer/probe targeting RU5
primer/probe targeting gag
G
Percentage of tRNA extension normalized to WT
140%
120%
100%
80%
60%
40%
20%
0
WT A140C G137C C173 G

Supplementary Figure 6. The mutations in the PBS-segment TLE lower stem did not affect $tRNA^{Lys3}$ annealing, *in vitro* primer extension, and $tRNA^{Lys3}$ placement on viral RNA. **A**. The NC-promoted RNA duplex formed between PBS-segment mutants and $tRNA^{Lys3}$ shifted to the same position as the WT PBS-segment:$tRNA^{Lys3}$. **B-E**. The *in vitro* RT primer extension products of Cy3-labled $tRNA^{Lys3}$ on PBS-segment (B), PBS-G137C (C), PBS-A140C (D), and PBS-C173C (E) were resolved on 10% denaturing polyacrylamide gels. **F**. To investigate $tRNA^{Lys3}$ placement on gRNA, RNA was extracted from vector viruses produced within 48 h post transfection. Virion RNA complexes were isolated and incubated with RNaseH deficient RT (SuperScript II). cDNA intermediates of tRNA annealing and extension by RT were detected by qPCR with primers complementary to RU5. The input gRNA amount was quantified by RT-qPCR of gag region. Negative control qPCR to amplify the gag region was also performed on viral RNA to establish minimal contamination from proviral DNA (gag DNA copy numbers were less than 1% of the RNA copy numbers measured by RT-qPCR). **G**. The amount of cDNA synthesized by extending the $tRNA^{Lys3}$ annealed on the mutant viral RNA was quantified and compared to that of the WT.

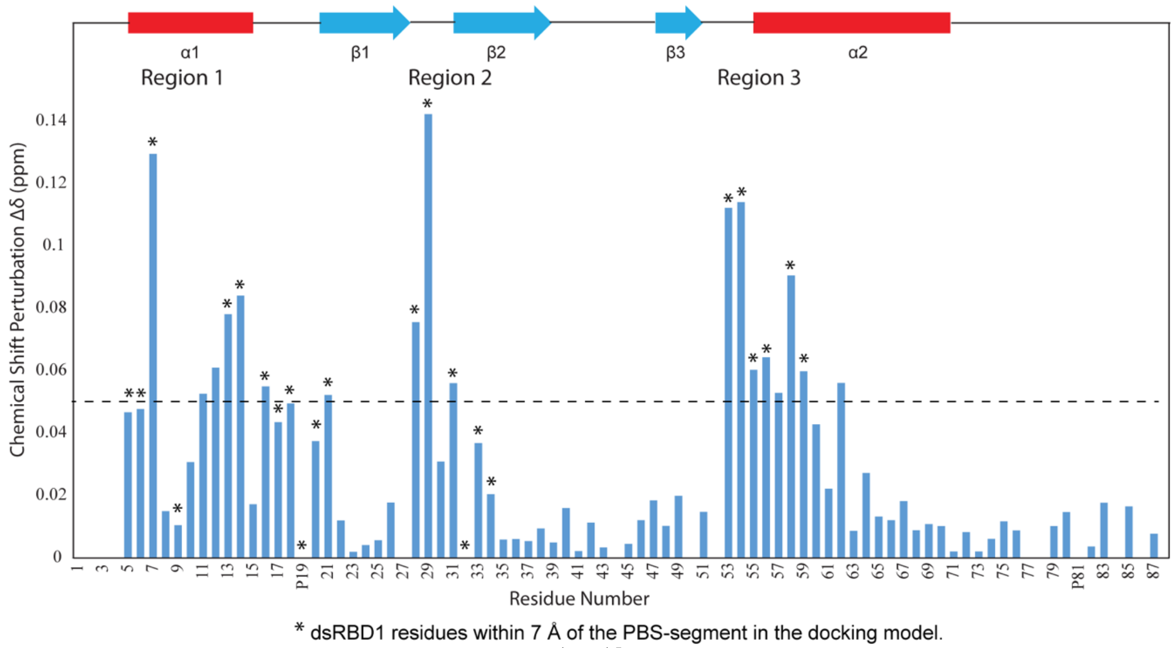

Supplementary Figure 7. CSPs of dsRBD1 ^{1}H-^{15}N backbone resonances upon the addition of PBS-segment RNA (the molar ratio of protein/RNA was 1:0.8) are plotted. Perturbations were calculated using the formula: $\Delta\delta = [\delta_{HN}^{2} + (\delta_{N}/6.51)^{2}]^{1/2}$ and plotted against the primary structure. Secondary structure elements based on the crystal structure of human RHA dsRBD1 (PDB: 3vyy) is shown on the top. No signals were detected for P19 or P81 as prolines do not have an amide proton. Amide protons of some residues, including G27, Q32, N50, S77 and E78, were not detected under the experimental conditions and thus their CSPs were not plotted. Asterisks (*) denote residues that are within 7 Å of the PBS-segment RNA in the docking model.

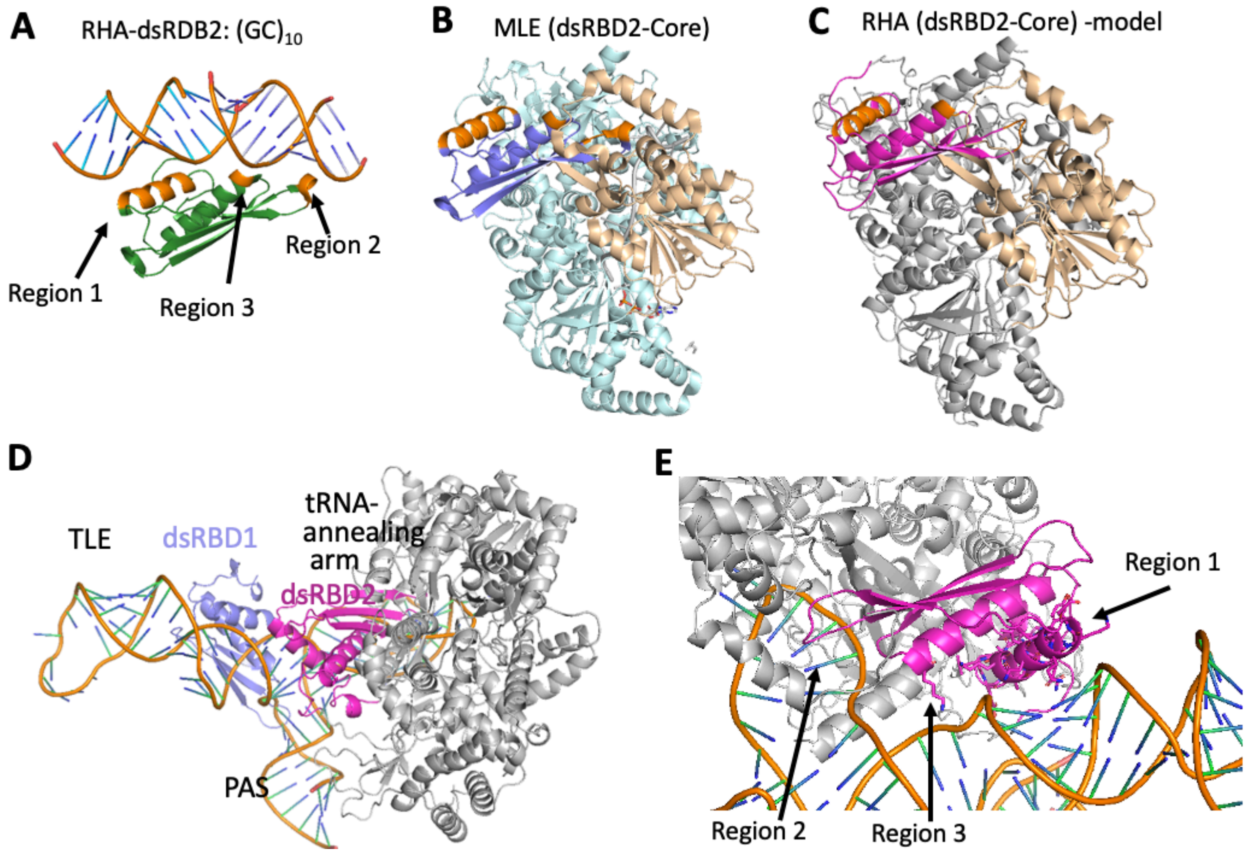

Supplementary Figure 8. A model of dsRBD2-Core docking onto PBS-segment presents possible interactions between RHA and PBS-segment. **A**. Cartoon view of RHA dsRBD2 (green) in complex with $(GC)_{10}$, with the RNA binding residues (regions 1-3) shown in orange (PDB: 3vyy). **B**. Cartoon view of MLE dsRBD2-Core. The dsRBD2 domain is shown in purple with the equivalent regions 1-3 shown in orange (PDB: 5aor). The RecA2 domain is shown in wheat. **C.** Cartoon view of RHA dsRBD2-Core model generated by homology modeling. The dsRBD2 domain is shown in magenta, and regions 1-3 are shown in orange. The RecA2 domain is shown in wheat. **D**. A model of RHA docked onto PBS-segment with the lowest energy score and does not interfere the dsRBD1 binding site. Purple: dsRBD1; pink, dsRBD2; grey, core domain of RHA. **E**. A zoom-in view of the dsRBD2:PBS interface. α1 (region1) binds to a minor groove in TLE lower stem and K236 (region 3) binds to the adjacent major groove. Residues in region 2 are not in the binding interface because it is surrounded by RecA2 domain.

Table S1. Primers for pNL4-3-CMV-EGFP mutagenesis

Mutants	Primers for Amplicon1	Primers for Amplicon2
A157G	BstZ17I-F; A157G-R	A157G-F; BstZ17I-R
ΔUC-Loop	BstZ17I-F; ΔUC-Loop-R	ΔUC-Loop-F; BstZ17I-R
G212C/U213C	BstZ17I-F; 212213-R	212213-F; BstZ17I-R
A214U	BstZ17I-F; A214U-R	A214U-F; BstZ17I-R
G137C	BstZ17I-F; G137C-R	G137C-F; BstZ17I-R
C173G	BstZ17I-F; C173G-R	C173G-F; BstZ17I-R
A140C	BstZ17I-F; A140C-R	A140C-F; BstZ17I-R

Table S2. Primer list for pNL4-3-CMV-EGFP mutagenesis

Primer	5'-3' sequence
BstZ17I-F	CCTTCACCTGAAATGTGTGTATACAAAATCTAGGCCAGTC
BstZ17I-R	CTAGGTATGGTAAATGCAGTATACTTCCTGAAGTCTTTATC
A157G-F	TTTGGTCAGTGTGGAAAATCTCTAGCAGTGGCGCCCGAAC
A157G-R	TCCACACTGACCAAAAGGGTCTGAGGGATCTCTAGTTACCAG
ΔUC-Loop-F	CAGAGGAGACTCAGTGTGGAAAATCTCTAGCAGTGGCGCCCGAAC
ΔUC-Loop-R	ACTGAGTCTCCTCTGAGGGATCTCTAGTTACCAGAGTCACACAACAG
212213-F	GCGAAACCAAAGCCAGAGGAGATCTCTCGACGCAG
212213-R	TGGCTTTGGTTTCGCTTTCAAGTCCCTGTTCG
A214U-F	GCGAAAGTTAAGCCAGAGGAGATCTCTCGACGCAG
A214U-R	TGGCTTAACTTTCGCTTTCAAGTCCCTGTTCG
G137C-F	GTAACTACAGATCCCTCAGACCCTTTTAGTCAGTGTGG
G137C-R	AGGGATCTGTAGTTACCAGAGTCACACAACAGACG
C173G-F	AATGTCTAGCAGTGGCGCCCGAACAG
C173G-R	CCACTGCTAGACATTTTCCACACTGACTAAAAGG
A140C-F	TGGTAACTAGAGCTCCCTCAGACCCTTTTAGTCAGTGTGGAAAATCTC
A140C-R	GAGCTCTAGTTACCAGAGTCACACAACAGACGGGCACACAC

Table S3. Primer list for quantitative PCR measurement

Primer		Sequence
KB2284	Early product sense	5’ GCC TCA ATA AAG CTT GCC TTG A 3’
KB2285	Early product antisense	5’ TGA CTA AAA GGG TCT GAG GGA TCT 3’
KB2363	Late product sense	5’ TGT GTG CCC GTC TGT TGT GT 3’
KB2364	Late product antisense	5’ GAG TCC TGC GTC GAG AGA TC 3’
KB3267	CCR5 sense	5’ CCA GAA GAG CTG AGA CAT CCG 3’
KB2368	CCR5 antisense	5’ GCC AAG CAG CTG AGA GGT TAC T 3’
RU5-F2	RU5 sense	5′ GCC TCA ATA AAG CTT GCC TTG A -3’
RU5-R3	RU5 antisense	5′ TAG AGT GGT CTG AGG GAT CT -3’
RU5-p	RU5 probe	5’ FAM-AGA GTC ACA CAA CAG ATG GGC ACA CAC T-TAMRA-3’
gag-F	gag sense	5’ CTA GAA CGA TT CGC AGT TAA TCC T 3’
gag-R	gag antisense	5’ CTA TCC TTT GAT GCA CAC AAT AGA G 3’
gag-P	gag probe	5’ FAM-CAT CAG AAG GCT GTA GAC AAA TAC TGG GA-TAMRA 3’

Table S4. SAXS statistics

Sample	PBS-segment
**a. Sample Details**	
Organism	HIV-1: pNL4-3
Source	NIH AIDS Reagent Program
Description	HIV-1 RNA pNL4-3 (125-223) synthesized with two terminal G-C pairs
Molecular masses from chemical composition (kDa)	33.3
SEC-SAXS column	GE Superdex 200 Increase 10/300
Loading concentration (mg/ml)	9
Injection volumn (µl)	200
Flow rate (ml/min)	0.75
Average concentration in combined data frames (mg/ml)	~ 2.2 (1.4 - 2.66)
Solvent composition	10 mM Tris, 140 mM KCl, 10 mM NaCl, 1 mM MgCl2, pH 7.5
**b. SAXS data collection parameters**	
Source and instrument	BioCAT 18ID beam line with Pilatus3 X 1M detector at the Advanced Photon Source (APS), Argonne National Laboratory storage ring (Lemont, IL, USA)
Wavelength (Å)	1.03
Sample-detector distance (m)	3.5
q-measurement range ($Å^{-1}$)	0.0054 – 0.3807
Exposure time (s) & number	876 successive 1 s frames
Sample temperature (°C)	25
**c.Software employed**	
SAXS data reduction	Beamline software
SAXS data Basic Analyses	PRIMUS/qt from ATSAS 2.8.0/3.0.0
Shape/bead modelling	DAMMIF from ATSAS 2.8.0
Atomistic modelling	CRYSOL from ATSAS 3.0.0
**d. Structural parameters**	
Guinier analysis	
I(0) (cm^{-1})	73.96 ± 0.11
R_g(Å)	33.59 ± 0.09
q-range ($Å^{-1}$)	0.0113 – 0.0386
$qR_g max$	1.3
Coefficient of correl. R2	0.997
P(r) Analysis	
I(0) (cm^{-1})	74.4
R_g (Å)	34.50
d_{max} (Å)	128
q-range ($Å^{-1}$)	0.0123 -0.238
GNOM total est.	0.81

M from I(0) (kDa)	40.0
**e. Shape model-fitting results**	
DAMMIF (default parameters, 10 calculations)	
q-range for fitting (Å^{-1})	0.0123 -0.238
Symmetry, anisotropy assumptions	P1, none
NSD (standard deviation)	0.731 (0.041)
χ^2 range	1.30 – 1.55
**f. Atomistic modelling**	
CRYSOL (with default parameters, No constant subtraction)	
χ^2,	1.40 ± 0.03
Predicted R_g (Å)	34.80 ± 1.69
Vol (Å),	32117 ± 334
Ra (Å),	1.45 ± 0.11
Dro (eÅ^{-3})	0.026 ± 0.003
**e. Data and model deposition ID**	
SASDB	SASDJU7

Table S5. ITC statistics for dsRBD1 binding to PBS-segment and hairpin control.

	PBS-segment	Hairpin-control
N1	0.9 ± 0.1	5.2 ± 0.2
K_{d1} (μM)	1.17 ± 0.23	18.5 ± 1.1
Δ*H* per site (cal/mol)	-7845 ± 123	-1762 ± 37
Δ*S* per site (cal/mol)	1.28 ± 0.08	-1.49 ± 0.10
N2	5.5 ± 1.3	
K_{d2} (μM)	45 ± 8.1	
Δ*H* per site (cal/mol)	-764 ± 88 [a]	
Δ*S* per site (cal/mol)	1.46 ± 0.23	

[a] The enthalpy change derived from the ITC data may be inaccurately estimated because the second set of binding sites are not saturated at the end of titration.
